# Chronic myeloid leukaemia cells require the bone morphogenic protein pathway for cell cycle progression and self-renewal

**DOI:** 10.1038/s41419-018-0905-2

**Published:** 2018-09-11

**Authors:** Parto Toofan, Caroline Busch, Heather Morrison, Stephen O’Brien, Heather Jørgensen, Mhairi Copland, Helen Wheadon

**Affiliations:** 1 Paul O’Gorman Leukaemia Research Centre, Institute of Cancer Sciences, College of Medical, Veterinary and Life Sciences, University of Glasgow, 21 Shelley Road, Glasgow, G12 0XB UK; 2 West of Scotland Genetic Department, QEUH, Glasgow, G51 4TF UK; 3 Northern Institute of Cancer Research, University of Newcastle, Newcastle, NE1 7RU UK

## Abstract

Leukaemic stem cell (LSC) persistence remains a major obstacle to curing chronic myeloid leukaemia (CML). The bone morphogenic protein (BMP) pathway is deregulated in CML, with altered expression and response to the BMP ligands shown to impact on LSC expansion and behaviour. In this study, we determined whether alterations in the BMP pathway gene signature had any predictive value for therapeutic response by profiling 60 CML samples at diagnosis from the UK SPIRIT2 trial and correlating the data to treatment response using the 18-month follow-up data. There was significant deregulation of several genes involved in the BMP pathway with *ACV1C, INHBA, SMAD7, SNAIL1* and *SMURF2* showing differential expression in relation to response. Therapeutic targeting of CML cells using BMP receptor inhibitors, in combination with tyrosine kinase inhibitor (TKI), indicate a synergistic mode of action. Furthermore, dual treatment resulted in altered cell cycle gene transcription and irreversible cell cycle arrest, along with increased apoptosis compared to single agents. Targeting CML CD34^+^ cells with BMP receptor inhibitors resulted in fewer cell divisions, reduced numbers of CD34^+^ cells and colony formation when compared to normal donor CD34^+^ cells, both in the presence and absence of BMP4. In an induced pluripotent stem cell (iPSC) model generated from CD34^+^ hematopoietic cells, we demonstrate altered cell cycle profiles and dynamics of ALK expression in CML-iPSCs in the presence and absence of BMP4 stimulation, when compared to normal iPSC. Moreover, dual targeting with TKI and BMP inhibitor prevented the self-renewal of CML-iPSC and increased meso-endodermal differentiation. These findings indicate that transformed stem cells may be more reliant on BMP signalling than normal stem cells. These changes offer a therapeutic window in CML, with intervention using BMP inhibitors in combination with TKI having the potential to target LSC self-renewal and improve long-term outcome for patients.

## Introduction

Chronic myeloid leukaemia (CML) treatment involves targeting BCR-ABL to prevent its tyrosine kinase activity. TKIs effectively target progenitor cells, however leukaemic stem cell (LSC) are more quiescent and less sensitive to treatment^1–5^. Studies of CML patients on imatinib mesylate (IM) treatment for >4 years indicate *BCR-ABL*^+^ cells are retained in the primitive CD34^+^ CD38^−^ population, even when a deep molecular response is achieved, thus CML LSC are not eradicated^6,7^. Recent clinical trials ‘The Stop Imatinib 1 (STIM1) and the STOP 2G-TKI Study’ are very encouraging with 38% and 43% of patients, respectively, sustaining long-term molecular responses when TKI was discontinued^8,9^. For many patients, TKI treatment alone is insufficient to cure CML, even when a sustained deep molecular response has been achieved, highlighting different pathophysiology for some patients.

Interest has therefore focused on developing new treatments to use in combination with TKI, thereby improving the number of patients who can discontinue treatment in the future without relapse. A feasible therapy approach would involve combining TKI with compounds to target alternative survival mechanisms, such as self-renewal pathways involved in HSC maintenance in the bone marrow (BM) niche and deregulated in CML^10–13^. These embryonic morphogenic pathways have a key role in hematopoiesis, with evidence suggesting LSCs hijack them to their advantage, making the BM microenvironment more suitable for their survival and proliferation^14^.

The TGFβ superfamily, including the bone morphogenic proteins (BMP) are important for sustaining BM homoeostasis. Transcriptome studies of CML LSCs and progenitors indicate that the TGFβ and BMP ligands are downregulated in chronic phase (CP)-CML, suggesting an extrinsic mechanism for TGFβ involvement in this disease^15^. Intriguingly, the BMP type I receptors, especially BMBR1B are overexpressed in CML LSCs, whereas *SMAD1*, *BMP2* and *BMP4* are downregulated^16^. Supporting our published microarray data^17^, which confirms that the BMP pathway and downstream signalling molecules are significantly deregulated in CP, accelerated phase (AP) and blast crisis (BC) CML in both primitive LSCs and progenitor subpopulations. These findings suggest CML LSCs may change their reliance/response to the BMP/TGFβ superfamily, especially as the disease progresses from CP to AP/BC^17^. This is supported by a study showing significantly higher levels of BMP2 and BMP4 ligands are present in CML patients’ BM, compared to normal donors. Moreover, CP-CML early progenitors express higher levels of type I receptors, making them more responsive to the increased levels of soluble BMP2 and BMP4 in the leukaemia BM niche, resulting in expansion. CML LSCs, when cultured in the presence of BMP2 or BMP4, maintained their primitive phenotype with enhanced long-term colony-forming potential^16^. LSCs from TKI-resistant patients also express higher levels of BMPR1B, BMP4 and *TWIST1* with treatment preferentially selecting survival of BMPR1B^Hi^ cells within the immature population. Mesenchymal stem cells (MSC) from these patients also displayed higher levels of BMP4 secretion^18^. These data indicate that alterations in the BMP pathway may suppress differentiation and potentiate the survival of a permanent autonomous pool of LSCs in CP-CML.

In this study, we evaluate the BMP pathway and downstream targets in 60 CP-CML patients at diagnosis. These findings were correlated to treatment response to identify a subset of genes differentially expressed between good/intermediate/poor responders to treatment. We demonstrate targeting the BMP receptors (ALKs) in combination with IM is synergistic, resulting in irreversible cell cycle arrest and increased apoptosis of CML cells. Furthermore, CML CD34^+^ cells display greater sensitivity to BMP pathway inhibition than normal CD34^+^ cells, undergoing fewer cell divisions, with reduced CD34^+^ cells numbers and colony formation occurring following treatment. Furthermore, CML-iPSCs express higher levels of ALKs than normal iPSCs and are more sensitive to ALK inhibition, resulting in a reduced capacity to self-renew. Overall, our findings indicate a potential therapeutic window whereby dual treatment with TKI and ALK inhibitors could selectively target CML stem cells.

## Results

### The BMP/SMAD pathway is deregulated in CP-CML

**Fig. 1:**
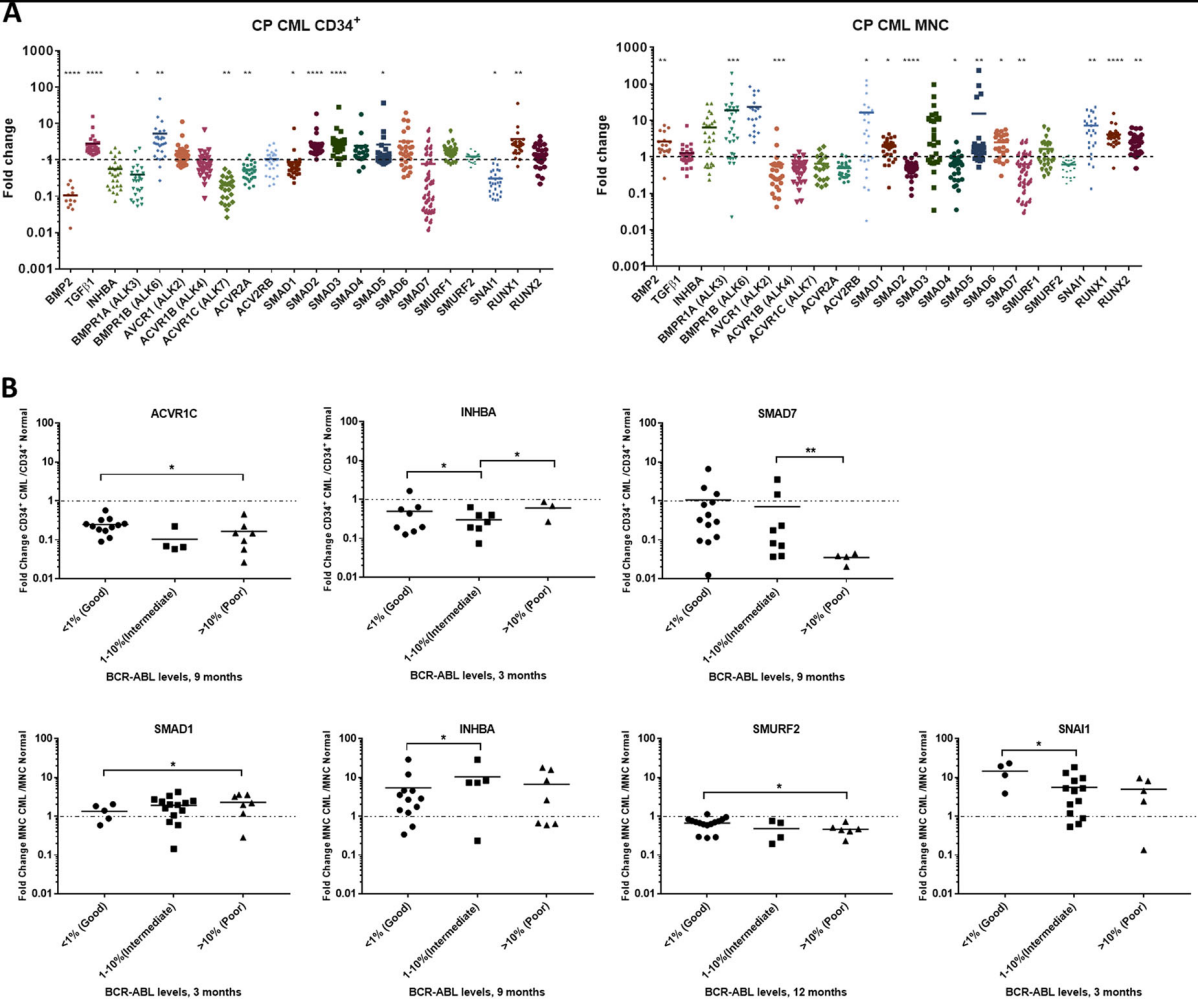
BMP pathway gene expression is deregulated in CP-CML and is associated with TKI response. **a** The BMP pathway and its downstream genes were analysed in 30 CP-CML CD34^+^ samples (stem/progenitor population) and 30 MNC CP-CML samples from the SPIRIT2 clinical trial using the Fluidigm Biomark system. Four normal CD34^+^ BM samples, two peripheral blood CD34^+^ normal donor samples and four normal MNC samples were used as comparators. Statistical analysis was performed using Welch’s test. **b** SPIRIT2 sample follow-up in CD34^+^ and MNC samples from ELN optimal, warning and failure patients. Analysis was performed by comparing the expression of each gene in poor, intermediate and good responders to the normal donors. Statistical analysis was performed using Student’s paired *t*-test. **p < 0.01–0.001, *p < 0.05–0.01

To characterise the BMP pathway, we analysed 60 CP-CML samples from the UK-based SPIRIT2 trial. A significant number of BMP-related genes were differentially expressed (Fig. 1a) in CML. Relative to normal controls, *BMP2*, *TGFβ1*, *AVCR1 (ALK2), BMPR1A (ALK3), SMAD1* and *SMAD2* showed opposite expression patterns when comparing the more primitive CML CD34^+^ population to the more mature MNCs. However, *ACVR1B (ALK4), BMPR1B (ALK6), ACVRIC (ALK7), ACVR2A*, *SMAD5 SMAD6, RUNX1* and *RUNX2* showed the same expression pattern in both populations. Using the 18-month follow-up data, patients were stratified into optimal, warning and treatment failure categories (termed “good/intermediate/poor TKI responders”) according to the European LeukemiaNet 2013 TKI response criteria^19^. We tracked gene expression patterns to clinical response, to identify a gene signature for TKI-responders vs non-responders (Fig. 1b and Table 1). In CD34^+^ samples, three genes *ACVR1C, INHBA*, *SMAD7*, and in MNC samples, four genes *SMAD1, INHBA, SMURF2* and *SNAIL1* showed significant differential expression in the good/intermediate/poor TKI responders. Interestingly, *INHBA* was the only gene upregulated in both the CD34^+^ and MNC intermediate/poor responders, this correlates with our previous data, indicating that *INHBA* is significantly upregulated in BC-CML LSC when compared to CP, and AP LSC, and normal HSC^17^ (GEO:GSE47927).

**Table 1:** Summary of statistical values for gene comparison between good/intermediate/poor TKI responders

*p*-Values CD34^+^					
Clinical monitoring		BCR-ABL	ACVR1C	INHBA	SMAD7
3 months	Good vs intermediate	<1% vs 1–10%	0.0458	0.9172	0.9506
	Good vs poor	<1% vs >10%	0.0518	0.0170	0.9222
	Intermediate vs poor	1–10% vs >10%	0.6752	0.0215	0.8824
	Good vs intermediate/poor	<1% vs >1%	0.0121	0.4609	0.9862
6 months	Good vs intermediate	<1% vs 1–10%	0.1489	0.2757	0.9659
	Good vs poor	<1% vs >10%	0.4402	0.5585	0.8917
	Intermediate vs poor	1–10% vs >10%	0.7035	0.4079	0.8788
	Good vs intermediate/poor	<1% vs >1%	0.0915	0.9304	0.9547
9 months	Good vs intermediate	<0.1% vs 0.1–1%	0.1982	0.3637	0.0004
	Good vs poor	<0.1% vs >1%	0.0206	0.8899	0.7088
	Intermediate vs poor	0.1–1% vs >1%	0.8047	0.4700	0.0260
	Good vs intermediate/poor	<0.1% vs >0.1%	0.0048	0.8645	0.1801
12 months	Good vs intermediate	<0.1% vs 0.1–1%	0.3331	0.9280	0.1183
	Good vs poor	<0.1% vs >1%	0.1792	0.8899	0.5539
	Intermediate vs poor	0.1–1% vs >1%	0.9487	0.8535	0.4452
	Good vs intermediate/poor	<0.1% vs >0.1%	0.0745	0.9179	0.1932
18 months	Good vs intermediate	<0.1% vs 0.1–1%	0.7984	0.9093	0.3802
	Good vs poor	<0.1% vs >1%	0.00005	0.00001	0.0074
	Intermediate vs poor	0.1–1% vs >1%	0.2686	0.0244	0.1939
	Good vs intermediate/poor	<0.1% vs >0.1%	0.1534	0.1755	0.8344

*p*-Values MNC						
Clinical monitoring		BCR-ABL	SMAD1	INHBA	SMURF2	SNAIL1
3 months	Good vs intermediate	<1% vs 1–10%	0.4601	0.4892	0.6416	0.0178
	Good vs poor	<1% vs >10%	0.0364	0.9185	0.3131	0.0912
	Intermediate vs poor	1–10% vs >10%	0.21	0.5518	0.1129	0.8693
	Good vs intermediate/poor	<1% vs >1%	0.1602	0.6435	0.8190	0.0114
6 months	Good vs intermediate	<1% vs 1–10%	0.9672	0.5779	0.0405	0.9394
	Good vs poor	<1% vs >10%	0.7407	0.0500	0.1476	0.0008
	Intermediate vs poor	1–10% vs >10%	0.7227	0.1227	0.2909	0.0568
	Good vs intermediate/poor	<1% vs >1%	0.9373	0.7969	0.0082	0.5516
9 months	Good vs intermediate	<0.1% vs 0.1–1%	0.9933	0.0294	0.1160	0.4770
	Good vs poor	<0.1% vs >1%	0.5802	0.9011	0.0382	0.7989
	Intermediate vs poor	0.1–1% vs >1%	0.4988	0.0827	0.6998	0.4280
	Good vs intermediate/poor	<0.1% vs >0.1%	0.7022	0.4699	0.0137	0.9539
12 months	Good vs intermediate	<0.1% vs 0.1–1%	0.2131	0.0001	0.4544	0.1290
	Good vs poor	<0.1% vs >1%	0.3529	0.7514	0.0227	0.5460
	Intermediate vs poor	0.1–1% vs >1%	0.0822	0.0281	0.8885	0.1894
	Good vs intermediate/poor	<0.1% vs >0.1%	0.9581	0.5950	0.0277	0.7893
18 months	Good vs intermediate/poor	<0.1% vs >0.1%	0.9690	0.6469	0.3967	0.6364

### ALK inhibitors act synergistically with IM

**Fig. 2:**
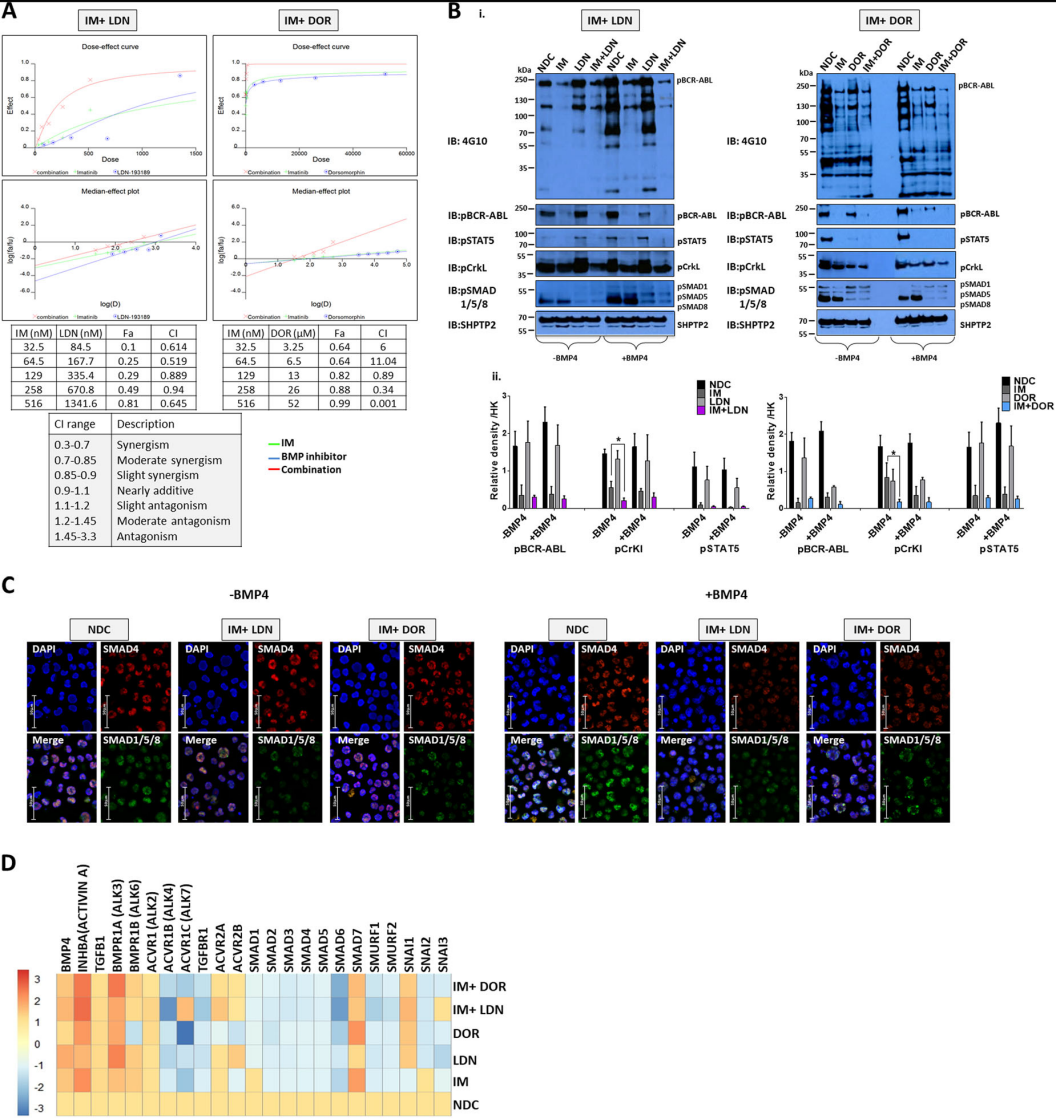
IM and BMP pathway inhibitors synergistically target the K562 CML cell line. **a** Synergy studies of the BMP pathway inhibitors, LDN-193189 (LDN) and Dorsomorphin (DOR) with imatinib mesylate (IM) were performed using CalcuSyn to quantify additive, synergistic and inhibitory effects. Tables present the fraction of cells affected (Fa) and combination index (CI) values at different combination concentrations. The bottom table describes the correlation between CI range and synergism. As shown, both LDN and DOR, in combination with IM, synergistically induce cell death in K562 cells. **b** Protein analysis of K562 cells treated with IM, BMP inhibitors and the combination (IM = 500 nM, LDN = 500 nM, DOR = 2.5 μM, *n* = 3) compared to no-drug control (NDC) using western blot hybridisation following drug treatment in the presence and absence of BMP4 stimulation. i IM and LDN combination immunoblots at 4 h (left panel) and IM and DOR combination immunoblots at 4 h (right panel). K562 cells were stimulated with 20 ng/mL BMP4 for 30 min; this was followed by drug treatment. SHP2 was used as the housekeeping protein. ii Densitometry analysis of western blots was performed using image J software. Analysis was normalised relative to the housekeeping protein expression. Both BMP inhibitors synergistically inhibit pCrKl significantly in the absence of BMP4 stimulation. Data are expressed as mean ± standard deviation and were compared using the unpaired Student’s *t*-test, *p < 0.05; *n* = 4. **c** Protein analysis using immunofluorescence in K562 cells treated with IM, BMP inhibitors and the combination of both (*n* = 3) at 24 h ± BMP4 stimulation. Panels of four pictures for each treatment include a single staining for SMAD1/5/8 in green, SMAD4 in red, the combination of both as the merge and no antibody in DAPI blue. **d** CD34^+^ primary CP-CML samples (*n* = 4) were treated with IM, BMP inhibitors and the combination of both (IM = 1 µM, LDN = 1 µM, DOR = 2.5 μM). Expression of BMP pathway genes was assessed at 72 h using the Fluidigm Biomark system; data were normalised to untreated cells

Next we inhibited BMP signalling using two ALK inhibitors, LDN and DOR^20,21^. IC50 values determined from XTT assays and trypan blue exclusion experiments were used to set up combination drug analyses with IM in K562 cells. Synergy calculations performed using CalcuSyn. Both LDN and DOR act synergistically with IM (Fig. 2a), with combination treatment more effective at eradicating leukaemic cells than single agents. Specificity of action was confirmed by immunoblotting (Fig. 2b) with dual inhibition significantly reducing phosphorylation of BCR-ABL, CrKL and STAT5. DOR and LDN both inhibited the BMP pathway with reduced pSMAD1/5/8 (R-SMADs) observed ±BMP4 (Fig. 2b, c); IM had no effect. Treatment of CD34^+^ CP-CML cells with LDN or DOR ±IM resulted in downregulation of BMP-related genes, especially the *SMAD* and *SMURF* family of genes. Interestingly *Activin A* and it’s receptor *BMPR1A (ALK3)* were upregulated following inhibition especially following IM/DOR treatment, this was accompanied by upregulation of *SMAD7* an Activin A inducible gene, which is a potent antagonist of TGFβ1 signalling (Fig. 2d).

### The impact of targeting the BMP pathway on the cell cycle

**Fig. 3:**
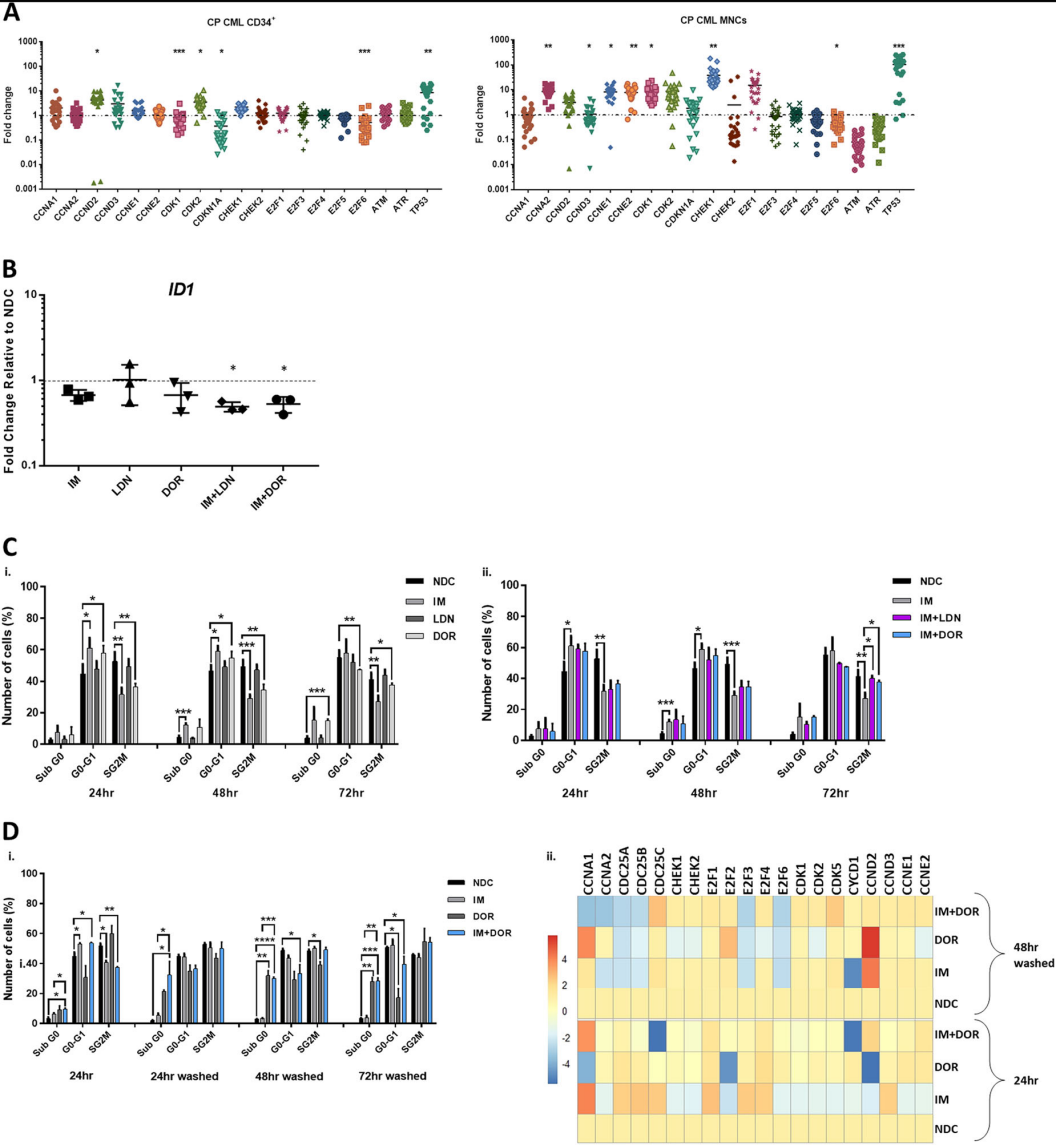

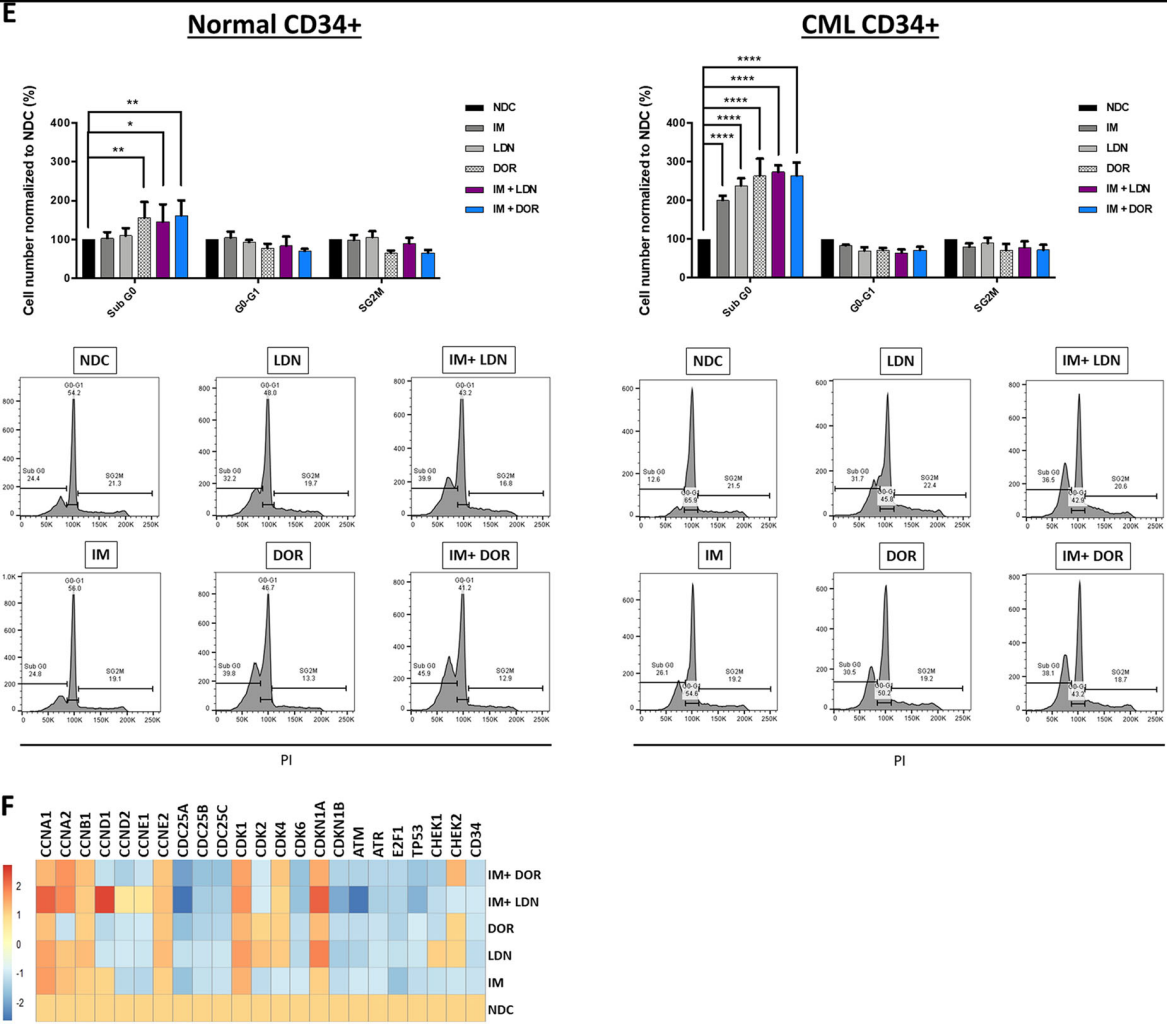
The effect of IM and BMP inhibitors on cell cycle progression and cell cycle gene expression pattern. **a** Cell cycle pathway gene expression of 60 patients enrolled in the SPIRIT2 clinical trial. Genes were analysed in 30 CP-CML CD34^+^ samples and 30 MNC CP-CML samples using the Fluidigm Biomark system. Four normal CD34^+^ BM samples, two peripheral blood CD34^+^ normal donor samples and four normal MNC samples were used as comparators. Statistical analysis was performed using Welch’s test. **b** The expression level of *ID* was assessed in CD34^+^ cells treated with IM, BMP inhibitors and the combination. Dual inhibition with both BMP inhibitors and IM decreased the expression of *ID* in all patient samples tested, compared to single-agent treatments. Statistical analysis was performed using Student’s paired *t*-test. *p < 0.05–0.01. **c** Propodium iodide (PI) analysis of cell cycle progression in K562 cells treated with IM, BMP inhibitors and the combination of both (IM = 500 nM, LDN = 500 nM, DOR = 2.5 μM, *n* = 4) after 72 h. i Treatment with individual inhibitors arrested cells in G1, with DOR causing more cell cycle arrest than LDN or IM. ii There is a further increase in the number of cells present in G1 phase when DOR was used in combination with IM (*n* = 4). **d-i** PI analysis of K562 cell cycle treated with IM, BMP inhibitors and the combination (IM = 500 nM, LDN = 500 nM, DOR = 2.5 μM, *n* = 3) when cells are washed to remove drugs 24 h post treatment. IM arrests K562 cells in G0-G1 at 24 h; however, cells go back into cycle after drug withdrawal. A different pattern was observed with DOR in combination with IM, where cells remained out of cycle after drug removal with a significant increase in the number of cells present in sub-G1. ii Expression analysis of cell cycle genes 48 h after drug wash-out, *n* = 3. **e** Cell cycle analysis of normal and CP-CML CD34^+^ samples treated with IM, BMP inhibitors and the combination of both (IM = 1 µM, LDN = 1 µM, DOR = 2.5 μM) at 72 h. Data show a significant increase in the number of cells in Sub-G0 in all single and dual treatments of normal and to a higher extent for CML samples (*n* = 3) (**f**) Analysis of cell cycle genes in CD34^+^ CP-CML cells in response to treatments (IM = 1 µM, LDN = 1 µM, DOR = 2.5 μM, *n* = 4). Cell cycle PI data are expressed as mean ± standard deviation and were compared using the unpaired Student’s *t-*test (**c**, **d**) or ANOVA (**e**). ****p < 0.0001, ***p < 0.001–0.0001, **p < 0.01–0.001, *p < 0.05–0.01

BCR-ABL is a potent driver of cell cycle, with IM causing G1 cell cycle arrest in CML. To ascertain the status of key genes involved in G1-S progression, we profiled the SPIRIT2 samples. Fig 3a clearly demonstrates important drivers of G1-S phase transition such as *CyclinE* and *CDK2* are upregulated in CP-CML. Interestingly, CML MNC express significantly higher levels of cell cycle genes than normal donors, indicative of the more immature/proliferative nature of the BCR-ABL-transformed cells. Using pathway analysis software, we mapped the links between BMP signalling and cell cycle in CML using gene profiling data (Figure S1).

BMP signalling upregulates the inhibitor of differentiation/DNA binding (ID) family of early response genes^22^. BCR-ABL also induces ID1 with high levels observed in CML^22,23^. *ID* expression is necessary for re-entry of quiescent cells into the cell cycle and G1-S progression^24^. Next, we ascertained whether treatment of CD34^+^ cells with IM alone and in combination with BMP inhibitors affected *ID1* expression. As shown, IM reduced the expression of *ID1* compared to NDC with expression further reduced in the combination approach (Fig. 3b). Cell cycle progression analysis showed DOR was more effective than LDN at perturbing progression in K562 cells (Fig. 3c). Treatment with IM or DOR arrested CML cells in G0-G1 phase, accompanied by a reduction in S/G2M (Fig. 3c–i). This increased with dual inhibition (Fig. 3c–ii). Assessment of cell cycle after dual IM/DOR treatment for 24 h followed by wash-out, indicates cell cycle arrest is irreversible (Fig. 3d–i). Gene profiling shows IM reduces the expression of *CDK* genes at 24 h with DOR having minimal effect at this time point (Fig. 3d–ii). Following treatment and wash-out, IM reduced the expression of *CCNA1*, *CCNA2*, *CDC25A*, *CDC25B*, *E2F3* and *E2F6* with dual inhibition (IM and DOR) further reducing expression (Fig. 3d–ii). Cell cycle analysis of CML CD34^+^ cells, revealed they were more sensitive to all treatment arms than normal CD34^+^, with a significant increase in cells in sub-G0 following dual treatment with LDN + IM, DOR alone and DOR + IM (Fig. 3e–ii). Similar results were obtained in the presence of BMP4 stimulation (Fig. S2A), with no changes when CD34^+^ were co-cultured on the stromal cell line HS5 (Figure S2B). Comparison of all the cell cycle data (Fig. S2C & D) indicates co-culture has a protective effect on CD34^+^ cell cycle arrest, preventing them from entering Sub-G0 following ALK inhibition. Important cell cycle genes also changed (Fig. 3f), with downregulation of *CDC25A*, *CDC25B* and *CDC25C*. *CDKN1B*, *CDK6, ATM and TP53* enhanced when IM and ALK inhibitors were combined.

### Dual inhibition results in increased apoptosis

**Fig. 4:**
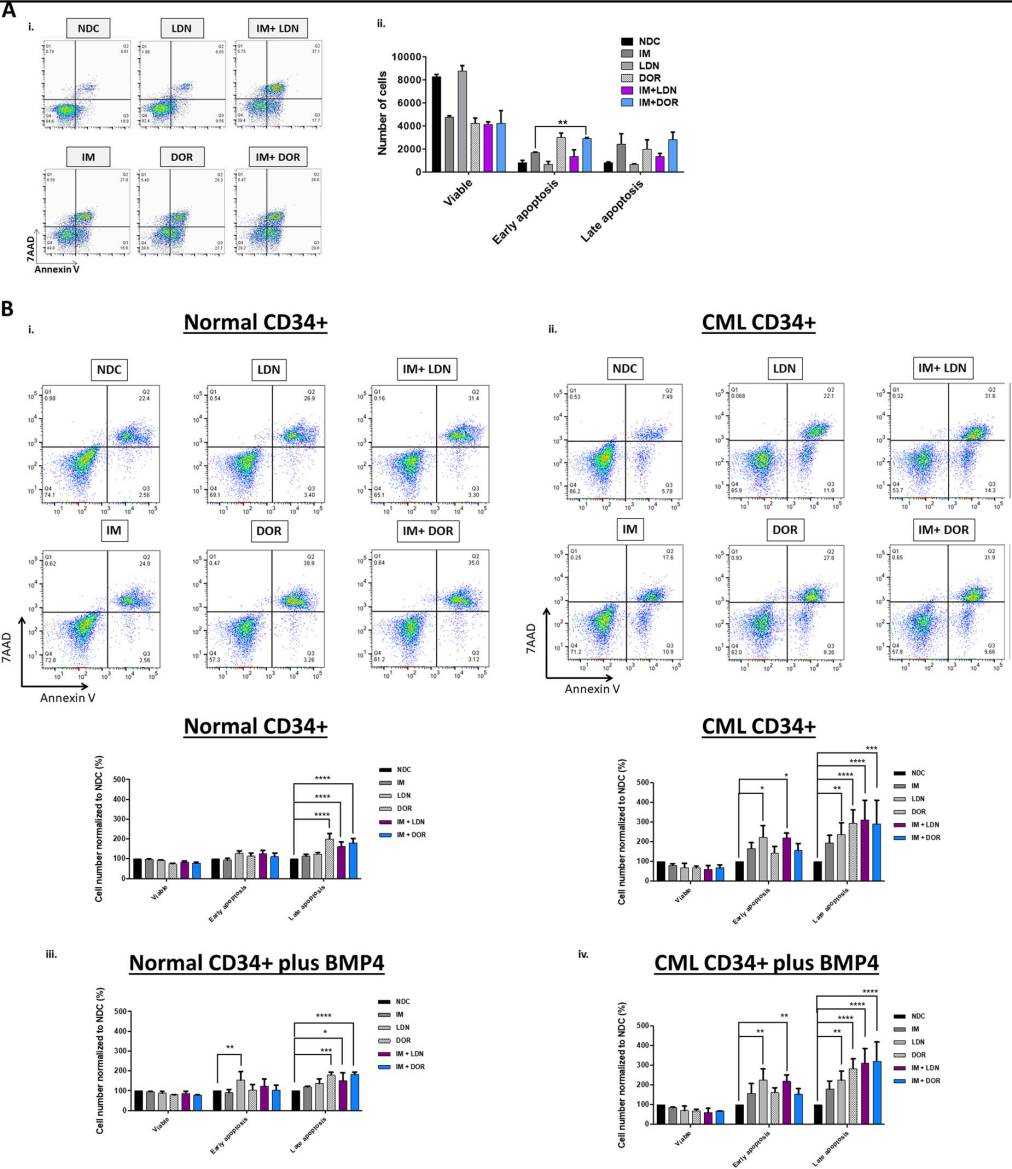
IM and BMP inhibitors induce apoptosis in CML cells. **a** Annexin V/ 7AAD apoptosis analysis of K562 cells treated with IM, BMP inhibitors and the combination (IM = 500 nM, LDN = 500 nM, DOR = 2.5 μM, *n* = 3) at 72 h. i Inhibition of BCR-ABL and BMP pathway results in apoptosis, and a reduction in viable cells. ii Results are significantly more profound when IM is used in combination with DOR with more cells in early apoptosis (*n* = 3). **b** Annexin V/7AAD apoptosis analysis of normal and CP-CML CD34^+^ cells treated with IM, BMP inhibitors and the combination (IM = 1 µM, LDN = 1 µM, DOR = 2.5 μM, *n* = 3) at 72 h. i, ii Results indicate that IM in combination with BMP pathway inhibitors promote modest, but significant, apoptosis in normal CD34 + cells. However, apoptosis is greatly increased in CP-CML CD34^+^ samples. This is reflected in the increased number of cells present in late apoptosis (*n* = 3). iii, iv Inhibitor treatments with addition of BMP4 also indicate a significant increase of normal and CP-CML CD34^+^ cells in late apoptosis, with more apoptosis observed in the CML CD34^+^ samples (*n* = 3)

To measure apoptosis, Annexin V/7AAD assays were performed in K562 (Fig. 4a), normal and CP-CML CD34^+^ cells (Fig. 4b). Inhibition of BCR-ABL and ALKs shifted CML cells into apoptosis. Results were more profound in IM + ALK inhibitor combination arms, with significantly higher levels of apoptosis observed in the CML CD34^+^ (Fig. 4b–ii, S3E) when compared to normal CD34^+^ cells (Fig. 4b–i, S3E). Similar results were observed in the presence of BMP4 stimulation (Fig. 4b–iii, iv & S3B). Co-culture on HS5 protected CD34^+^ cells from undergoing apoptosis in response to treatment (Fig. S3C–E). Higher levels of apoptosis occurred at 72 h in the NDC for normal and CML CD34^+^ cells co-cultured on HS5 (Fig. S3C–E), this is due to the increased proliferation observed, with on average a twofold expansion of cell numbers when compared to culture on plastic ±BMP4 at 72 h.

### ALK inhibition reduces CML CD34^+^ cell division and numbers

**Fig. 5:**
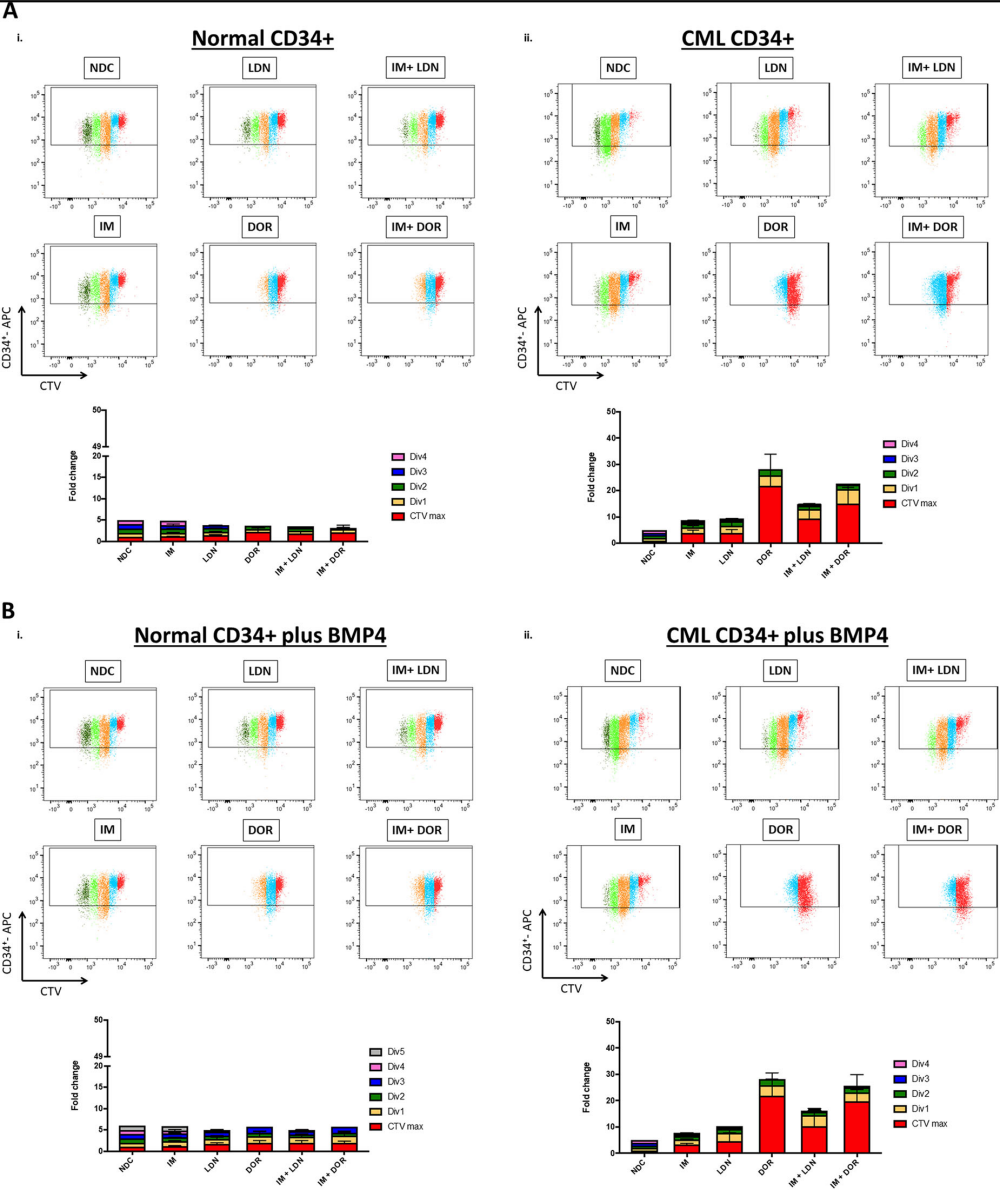

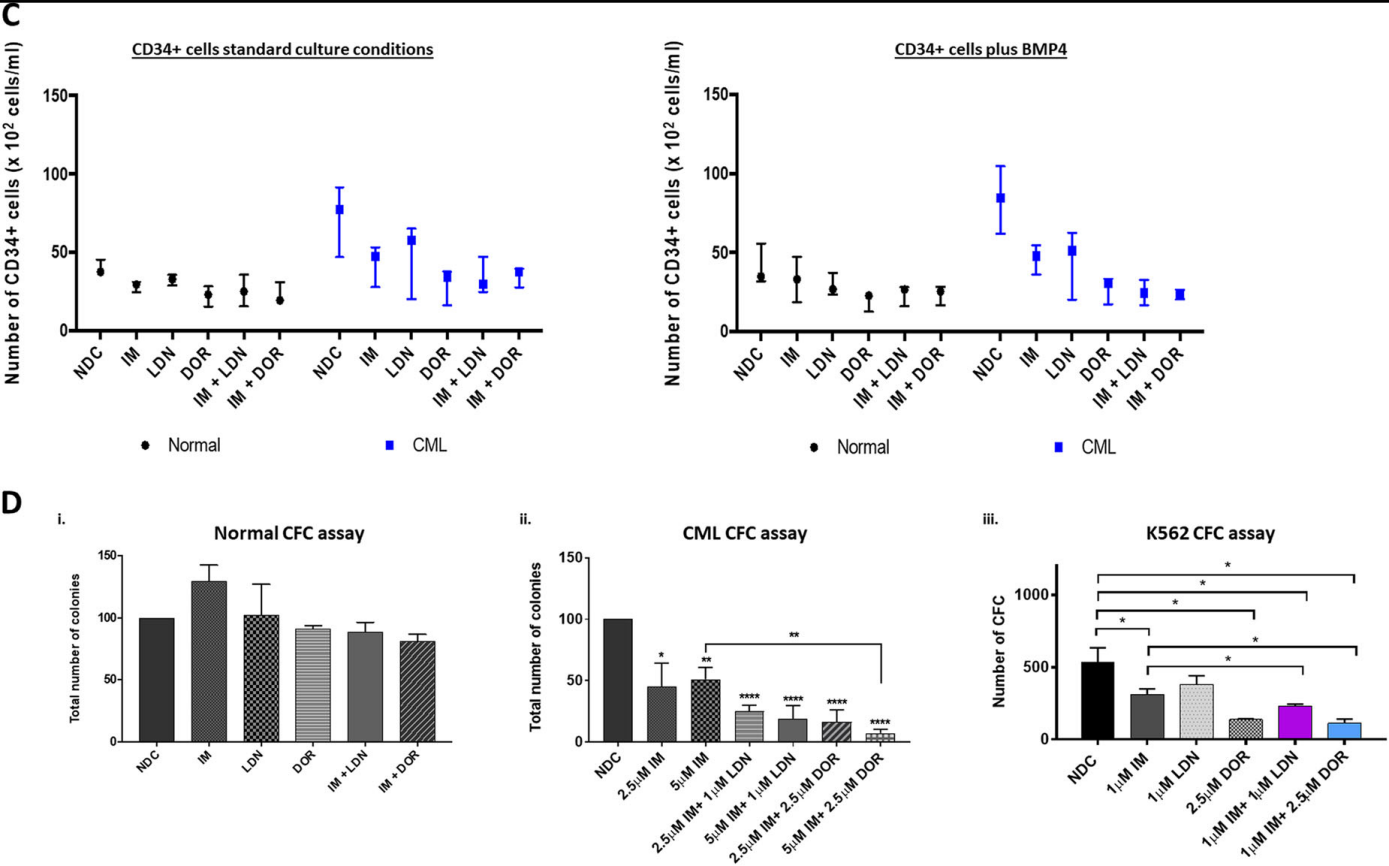
Single treatment of DOR and dual treatments of BMP inhibitors and IM inhibit cell proliferation of CML CD34+ cells. **a**, **b** CellTrace™ Violet (CTV) proliferation analysis of normal and CP-CML CD34^+^ cells treated with IM, BMP inhibitors, the combination (IM = 1μM, LDN = 1 μM, DOR = 2.5 μM, *n* = 3), in absence or presence of BMP4 at 72 h. i Results for normal CD34^+^ cells display that single and dual treatment of IM + DOR inhibit cell proliferation, and additional BMP4 treatment (**b**) results in more cell divisions compared to standard culture conditions without BMP4. **a-ii** Proliferation analysis of CP-CML samples indicates that LDN in combination with IM synergistically inhibits proliferation compared to single treatment. DOR alone and in combination with IM displays the biggest fold change compared to NDC. Additional treatment with BMP4 (**b-ii**) did not reveal differences in proliferation progression compared to standard culture conditions. **c** IM and BMP inhibitors reduce CD34^+^ cell numbers in CP-CML samples in absence or presence of BMP4. Results are displayed as median range of CD34^+^ cell numbers of normal and CP-CML CD34^+^ cells treated with IM, BMP inhibitors and the combination (IM = 1 µM, LDN = 1 µM, DOR = 2.5 μM, *n* = 3) in absence or presence of BMP4. **d-i** CFC assay results of normal CD34^+^ cells treated with IM, BMP inhibitors and the combination of (IM = 1 µM, LDN = 1 µM, DOR = 2.5 μM) do not reveal any significant difference compared to NDC (*n* = 3). ii Total number of colonies of CP-CML CD34^+^ cells display a significant difference across all treatments compared to NDC and a synergistic reduction when IM and DOR were used in combination (*n* = 3). iii CFC results of K562 cells. Results also indicate a significant drop in the number of colonies with IM, DOR and LDN. Results are also reflected in the morphology of the colonies with DOR and LDN treatment reducing the size of colonies (*n* = 3). Data are expressed as mean ± standard deviation and were compared using ANOVA (**c**) and the unpaired Student’s *t*-test (**a**, **b**, **d**), ***p < 0.001–0.0001, **p < 0.01–0.001, *p < 0.05–0.01

To determine what effects BMP pathway inhibition had on cell division and overall CD34^+^ cell numbers, CD34^+^ cells were labelled with CellTrace™ Violet (CTV) and an APC-conjugated anti-CD34 antibody. Following 72 h drug treatment, proliferation and CD34-positivity were assessed by flow cytometry. Figure 5a & S4B clearly demonstrate that both normal and CML CD34^+^ cells are sensitive to DOR treatment, undergoing less cell divisions than NDC. CML CD34^+^ cells displayed enhanced sensitivity, responded to all treatment arms with more cells accumulating in CTV^max^ and early divisions than observed for normal CD34^+^, with the most dramatic effects observed within the DOR, IM + LDN and IM + DOR treatment arms (Fig. 5a–ii and S4B). Similar results were observed in the presence of BMP4 stimulation (Fig. 5b and S4C). Although BMP4 stimulation did not alter the capacity to divide, untreated CML cells underwent a 20.5% ± 12.6% (*n* = 3) expansion in CD34^+^ numbers following BMP4 stimulation, with no change observed in normal CD34^+^ numbers (Figure S4E–iii), confirming CML cells are more responsive to BMPs^16^. Co-culture on HS5 rendered the drugs less effective, however CML cells still accumulated in CTV^max^ and division 1 following DOR, IM + LDN and IM + DOR treatment, indicating the BMP inhibitors were still eliciting an effect albeit to a reduced capacity (Figure S4A & D). Figure 5c and S4E clearly demonstrate that IM, LDN and DOR lead to a reduction in CML CD34^+^ cells, with the biggest reduction observed following DOR, IM + LDN and IM + DOR treatment. To determine what effects inhibition had on CML cell expansion and differentiation along the different hematopoietic lineages, CFC assays were performed. In CML, CFCs were significantly decreased in all treatment arms with less colonies observed following combination treatment (Fig. 5d–ii, iii) with no significant effects on normal CD34^+^ (Fig. 5d–i). These findings indicate viable CML cells surviving 72 h of dual treatment are unable to proliferate and differentiate to the same capacity compared with IM alone, indicating BMP inhibitors may act synergistically with IM to target CML cells through irreversible cell cycle arrest and apoptosis.

### CML-iPSC are reliant on the BMP pathway for their self-renewal

**Fig. 6:**
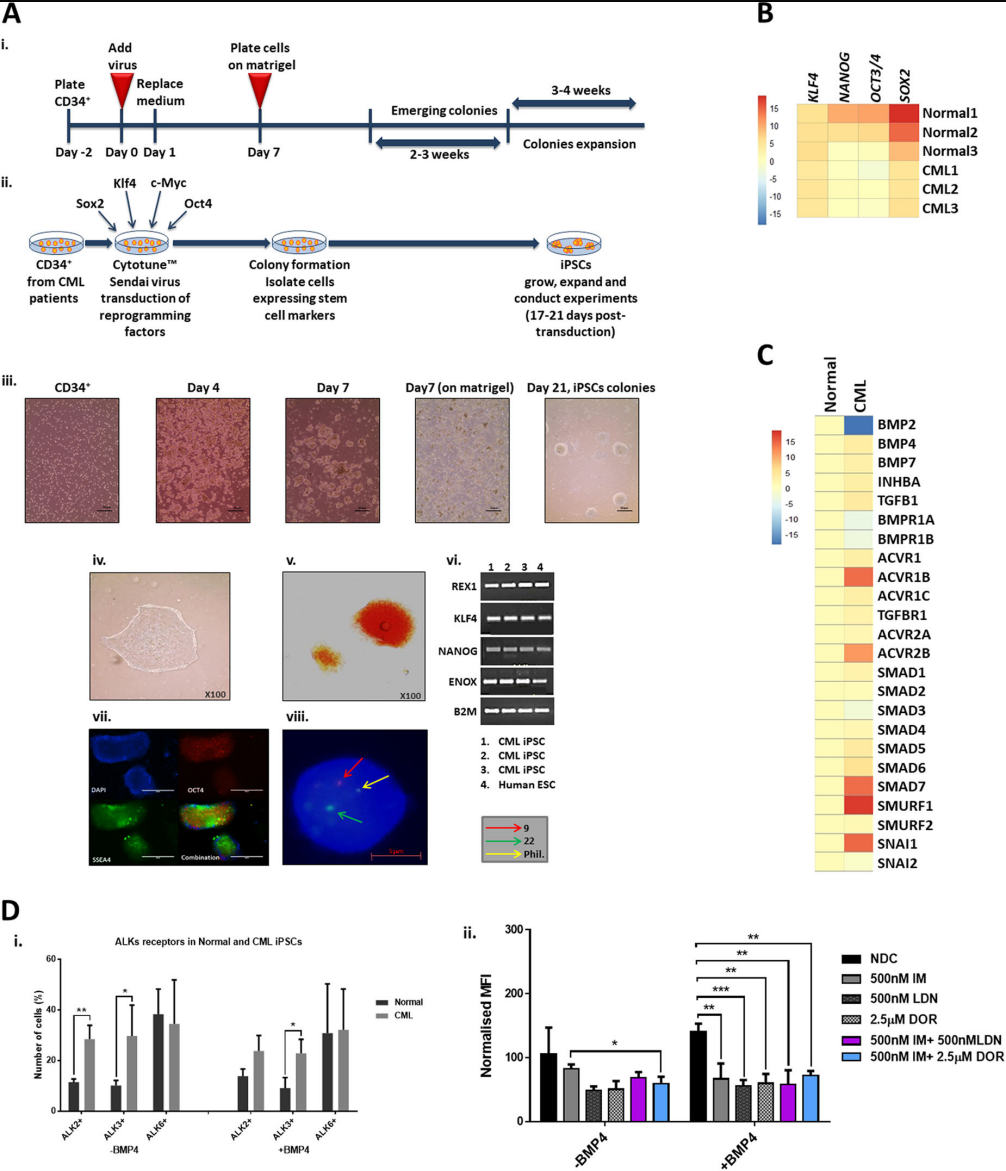

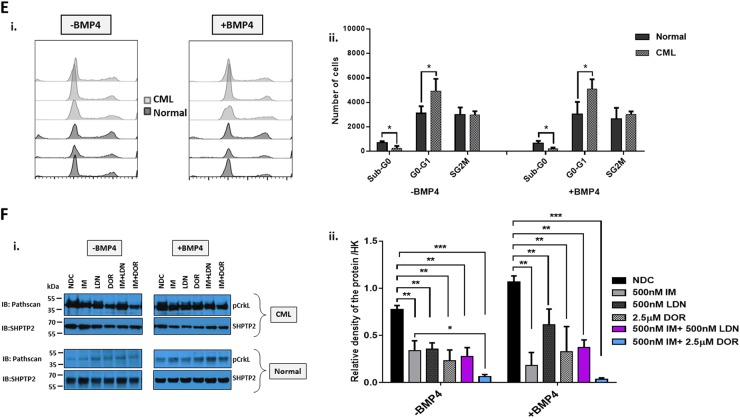
Deregulation of the BMP pathway in CML-iPSC. **a** Schematic representation of generating iPSCs from treatment naive CP-CML and normal samples using Sendai virus transduction of the reprogramming genes. i Experiment timeline for reprogramming CD34^+^ cells. ii A schematic representation of Sendai virus reprogramming of CD34^+^ cells from CP-CML patients. iii The morphology of cells during reprogramming period from single cells in suspension to iPSC colonies attached to matrigel coated plates. iv An iPSC colony with defined edges and a compact core. iPSCs were regularly passaged and colonies with highest pluripotency quality were plucked to help maintain this morphology. v Alkaline phosphatase (APh) staining. vi The expression level of *REX1*, *KLF4* and *NANOG* were assessed by PCR in all iPSCs. vii Immunofluorescence staining with TRA1-60, SOX2, OCT4 and SSEA4 was used to confirm pluripotency at protein level in iPSCs. viii Fluorescent in situ hybridisation (FISH) confirming the presence of BCR-ABL translocation in CML-iPSC colonies. **b** qRT-PCR confirming the expression of important pluripoptency genes in the three normal and three CML-iPSC samples. **c** The expression level of BMP pathway components analysed using qRT-PCR in CML-iPSCs. Data were normalised relative to normal-iPCSs. **d-i** Analysis of BMPRIs (ALK2, 3 and 6) in normal and CML-iPSCs using flow cytometry. To stimulate BMP pathway, cells were treated with 20 ng/mL BMP4 for 24 h prior to experiment. CML-iPSCs express significantly higher levels of ALKs 2 and 3 in the presence and absence of BMP4 stimulation compared to normal samples. ii FACS analysis of ALK2 expression in CML and normal iPSCs following drug treatment. Results are expressed relative to NDC. **e** Cell cycle analysis of normal and CML-iPSCs in the presence and absence of BMP4 stimulation. **f-i** Protein analysis of pCrKL in normal and CML-iPSCs treated with IM, BMP inhibitors and the combination of both using immunoblotting ± BMP4 stimulation at 4 h. SHPTP2 was used as a control. ii Statistical analysis of immunoblots shows that dual inhibition using IM and DOR has a synergistic effect on reducing pCrKl levels in CML-iPSCs. Data are expressed as mean ± standard deviation and were compared using Anova and the unpaired Student *t*-test, ***p < 0.001–0.0001, **p < 0.01–0.001, *p < 0.05–0.01, *n* = 3

To investigate whether CML stem cells are more dependent on the BMP pathway than their normal counterparts, we utilised iPSC models. We generated iPSCs from CD34^+^ selected cells from normal donors and CP-CML patients (Fig. 6a). All six lines demonstrate typical characteristics of pluripotent stem cells; compact morphology, strong alkaline phosphatase (APh) activity, high expression of key pluripotency transcription factors and pluripotent cell surface markers. FISH analysis confirmed the presence of the Philadelphia chromosome in the three CML-iPSCs lines (Fig. 6a, b). Similar to CML CD34^+^ cells, gene profiling indicate components of the BMP pathway were differentially expressed in CML-iPSCs compared to normal iPSCs, with *BMP2* strongly downregulated and *SMURF1* strongly upregulated (Fig. 6c). To check the ability of normal and CML-iPSCs to respond to BMP ligands, we analysed AVCR1 (ALK2), BMPR1A (ALK3) and BMPR1B (ALK6) expression ±BMP4 stimulation by FACS. CML-iPSCs expressed significantly higher levels of AVCR1 (ALK2) and BMPR1A (ALK3) receptors than normal iPSCs, with high levels of BMPR1B (ALK6) expressed by both (Fig. 6d–i). Treatment with IM, BMP inhibitors or in combination, ±BMP4 stimulation, resulted in a significant reduction in AVCR1 (ALK2) expression (Fig. 6d–ii), with no changes in BMPR1A (ALK3) and BMPR1B (ALK6) (data not shown) in CML-iPSCs. Treatment had no effect on normal iPSCs (data not shown). Cell cycle analysis revealed altered profiles in CML-iPSCs compared to normal iPSCs with fewer cells in the sub-G0 stage and more in G0-G1 ±BMP4 stimulation (Fig. 6e). BMP signalling is important for controlling cell cycle, we therefore assessed the effects of single and dual treatments on cell cycle, ±BMP4 stimulation in our iPSC model of CML. Following 72 h treatment, no significant changes in the cell cycle profile occurred regardless of drug treatment or BMP4 stimulation (Figure S5A). To assess signalling pathways affected by BCR-ABL, we monitored the phosphorylation of pCrkL by immunoblotting following 4 h of treatment ±BMP4 stimulation in normal and CML-iPSCs (Fig. 6f–i). As expected, normal iPSCs have very low levels of CrkL with no change in phosphorylation observed following drug treatments or BMP4 stimulation. Whereas CML-iPSCs had high levels of pCrkL, which was significantly reduced following IM and DOR treatment. Densitometry analysis indicated IM + DOR had a synergistic effect on reducing pCrkL levels (Fig. 6f–ii).

**Fig. 7:**
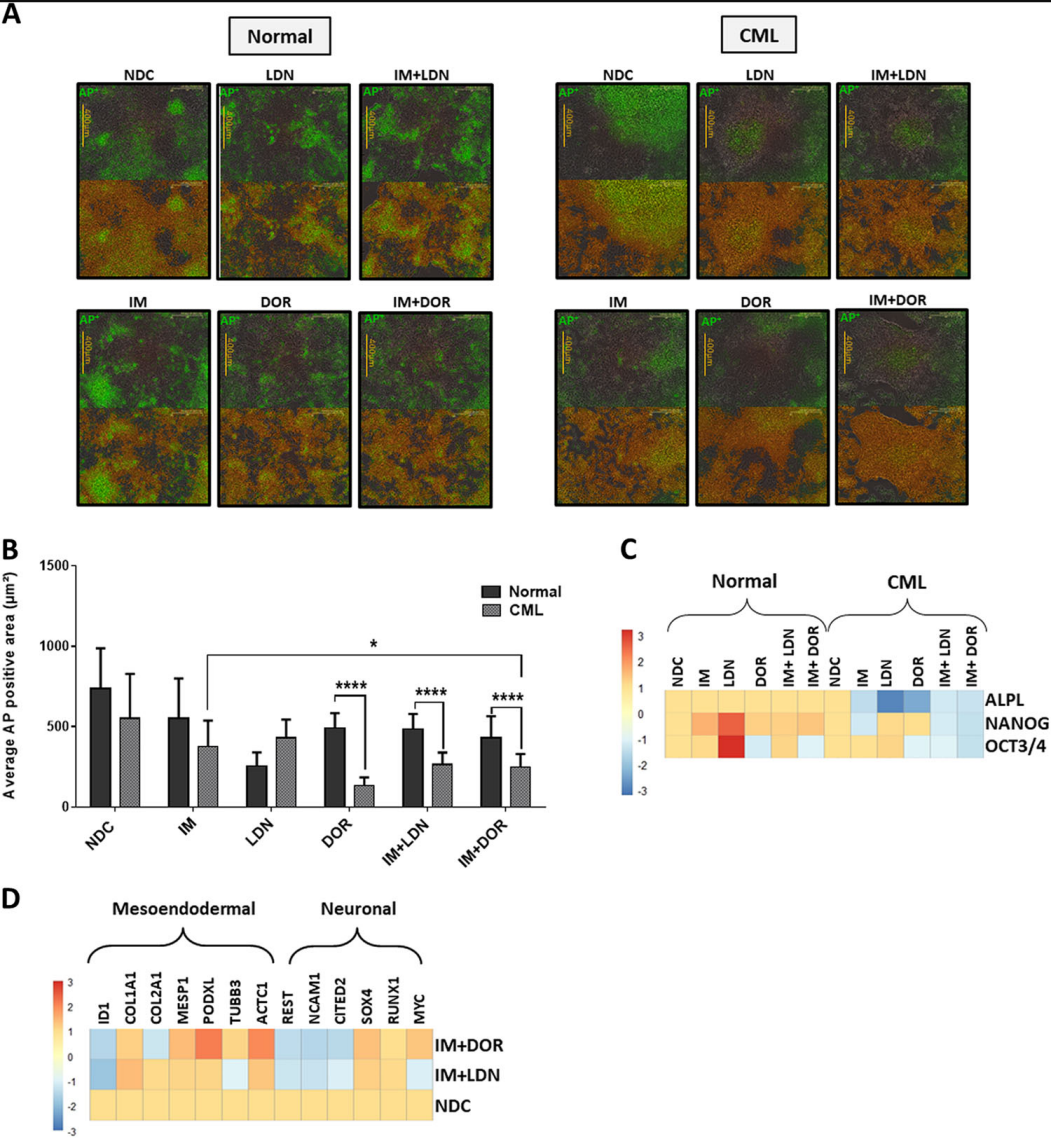

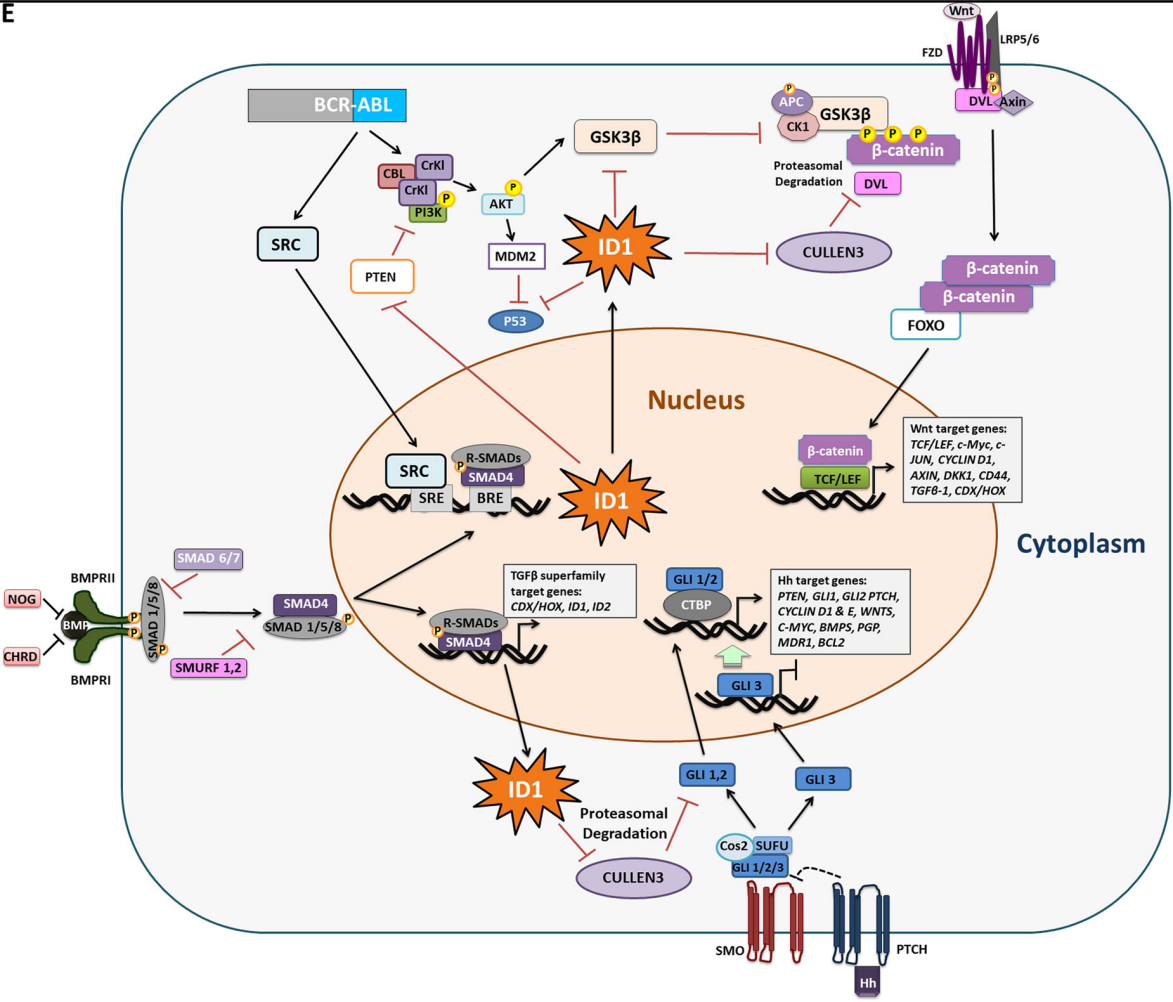
Effect of inhibiting the BMP pathway on CML-iPSC self-renewal. **a** Monitoring the pluripotency level of normal (left panel) and CML (right panel) iPSCs treated with IM, BMP inhibitors or the combination of both (IM = 1 µM, LDN = 1 µM, DOR = 2.5 µM) at 72 h using live APh staining. Pictures were taken by IncuCyte live imaging system. Green fluorescent tag illustrates APh positive cells. Orange fluorescent tag stains all of the colonies. **b** Comparison of surface area expressing APh in CML-iPSCs vs normal. Untreated sample was used as a comparator. Data are expressed as mean ± standard deviation and were compared using Anova and the unpaired Student’s *t*-test, ***p < 0.001–0.0001, **p < 0.01–0.001, *p < 0.05–0.01, *n* = 28 pictures for each arm. **c** Gene expression analysis shows a marked reduction in the expression of *ALPL*, *NANOG* and *OCT3/4* in CML samples only. **d** The expression of early differentiation genes assessed in CML-iPSCs when IM is used in combination with either BMP inhibitor. Data normalised with untreated samples. **e** Schematic diagram demonstrating the potential role of ID1 in CML pathophysiology. Embryonic morphogenic pathways and their downstream targets play key roles in the pathophysiology of CML, the early response gene *ID1* could be an important orchestrator in this process. *ID1* upregulation occurs through BCR-ABL-dependent and independent mechanisms. *ID1* expression is enhanced through BCR-ABL-mediated STAT5 and SRC activation, with the *ID1* promoter having a SRC-responsive element upstream of the translational start site. BMP/SMAD signalling regulates *ID1* through BRE in its promoter. Therefore, SRC can co-operate with SMAD to induce *ID1* expression. Once expressed, ID1 is known to mediate its effects by downregulating *p53* and *PTEN* transcription, resulting in enhanced AKT phosphorylation and AKT-mediated canonical Wnt signalling. ID1 can also enhance G1-S cell cycle progression and augment Hh and Wnt signalling through suppression of CULLIN3, an ubiquitin ligase which targets CyclinE, GLI2 and DVL2 for degradation. BMP and Wnt pathways also converge to regulate the CDX family of homeobox transcription factors, master regulators of *HOX* gene expression, important transcription factors involved in CML

A fundamental property of iPSCs is their ability to self-renew. Live APh staining was used to monitor pluripotent state in CML- and normal iPSCs treated with single and dual therapies for 72 h (Fig. 7a). APh stain intensity (green) was compared to total stain (orange); NDC sample was used as a comparator in each set. CML-iPSCs differentiated more than normal iPSCs when treated with IM and ALK inhibitors. This effect was more evident when colonies were treated with IM alone or in combination with DOR (Fig. 7b) especially following longer drug treatments (Figure S5B–i, ii) with dual inhibition more effective at reducing the pluripotency of CML-iPSCs. Single and dual inhibition also caused reduction in the expression of important pluripotency genes in CML colonies only, with all the genes assessed reduced by dual inhibition (Fig. 7c). Analysis of key differentiation genes indicate cells are primed to undergo meso-endodermal differentiation with *ACTC1*, *MESP1* and *PODXL* all being upregulated following dual inhibition of CML-iPSCs (Fig. 7d). Overall these results indicate that CML-iPSC depend more on BMP signalling to sustain their stem cell properties than normal iPSC.

## Discussion

In CML, minimal residual disease persists in many patients on TKIs, with a high level of relapse occurring if TKI is withdrawn^8,9^. Therefore, additional kinase-independent mechanisms involved in LSC persistence need to be identified and targeted if treatment-free remission is to be achieved. Recent evidence indicates that some of the important regulatory networks involved in establishing primitive and definitive hematopoiesis during development are reactivated in leukaemias, giving rise to a LSC population with altered self-renewal and differentiation properties^25^. One pathway warranting further investigation is BMP signalling. BMPs are fundamentally important for osteoblast differentiation^26,27^, self-renewal and maintenance of HSCs, and expansion and differentiation of progenitor cells into mature hematopoietic cells^28–31^. Previously, we and others highlighted that the BMP pathway was deregulated in CML^15,17^. Furthermore, an earlier study demonstrated higher than normal levels of BMP ligands in the BM of CML patients and enhanced expression of BMPR1B receptor by CP-CML CD34^+^, with BMP2 or BMP4 stimulation, maintaining their primitive phenotype^16^. Data from the same group demonstrated that high BMPR1B and *TWIST1* expression is linked to treatment resistance and disease progression^32^, with treatment resulting in upregulation of *TWIST1* and selecting for survival of BMPR1B^Hi^ cells^18^. Furthermore, the LSC and MSC populations from these patients expressed higher levels of BMP4. In LTC-IC assays performed on BMPR1B^Hi^ and BMPR1B^Lo^ LSC, more LTC-IC were present in the presence of BMP2/4 and following IM and IM/IFNα treatment in the BMPR1B^Hi^ LSC, indicating the BMP signalling pathway has an important role in LSC maintenance and CML disease persistence^18^.

Components of the BMP pathway were significantly deregulated in both CD34^+^ cells and MNCs from CP-CML SPIRIT2 samples. In CD34^+^ cells, *BMP2* was downregulated, whereas type 1 receptors *AVCR1 (ALK2), BMPR1B (ALK6), TGFβΡ1* and several of the *SMAD* genes were upregulated. In contrast, *BMP2* transcript was upregulated in MNC along with *BMPR1A (ALK3), BMPR1B (ALK6)* and *SMADs*. These findings support previous findings showing BMP2 is produced by the mature CML polymorphonuclear cells, and CML CD34^+^ cells are likely to have potentiated signalling in response to ligand^16^. TGFβ signalling is linked to poor TKI response in CML, with enrichment of the pathway observed at the single-cell level in poorly responding BCR-ABL^−^ and BCR-ABL^+^ CML cells^33^. TGFβ signals through Foxo3A to confer apoptosis resistance in CML LSC^34^. Also TGFα is a potential biomarker for predicting patient response to TKI, with high levels in patient serum at diagnosis correlating to a failure to obtain a major molecular response^35^.

Correlating our expression data to TKI response, three genes (*ACVR1C, INHBA* and *SMAD7*) in the CD34^+^ samples, and four genes (*SMAD1, INHBA, SMURF2* and *SNAIL1*) in the MNC samples, showed significant differential expression between the good/intermediate/poor responders. *INHBA* was more highly expressed in the poor response group, and is highly expressed in BC-CML^17^. Treatment of K562 with IM and the BMP pathway inhibitors also resulted in upregulation of *Activin A* and its receptor along with *SMAD7* an Activin A inducible gene, which is a potent TGFβ1 antagonist. Interestingly, high levels of *INHBA*, correlates inversely with survival in solid tumours^36,37^ and is linked to immunosuppression^38^. In multiple myeloma, high levels of Activin A correlates with disease progression due to its role in BM niche remodelling through osteolysis^39^. Activin A via non-canonical SMAD2 signalling can also activate β-catenin leading to upregulation of the TCF/LEF transcriptional regulators^38^, a pathway well described in CML progression and TKI resistance^40^. These findings indicate an important role for BMP/TGFβ superfamily in the pathogenesis of CML and a potential switch from BMP to Activin A signalling in treatment resistance and progressive disease.

Cell cycle deregulation is well documented in CML^41–43^, with BMP signalling important for G1-S progression^44^. Targeting the BMP pathway in combination with TKI in CML resulted in cell cycle arrest, apoptosis and altered target gene expression. Moreover, cell division analysis revealed that DOR prevents CML CD34^+^ from dividing and reduces the total number of CD34^+^ cells, with similar results observed in the IM + DOR treatment arm. Total CFC counts also illustrate that the combination treatment had a more potent effect on long-term CML cell survival and colony formation than single-agent treatment. Analysis of normal CD34^+^ cells revealed significantly less sensitivity to the BMP pathway inhibition, indicating a potential therapeutic window to target this pathway in CML. Importantly, given the high BMP4 concentrations in the BM, inhibition of the pathway still occurred following BMP4 stimulation using both BMP pathway inhibitors.

We generated iPSCs as a stem cell model, the rationale for this approach was that pluripotent stem cells rely on the BMP pathway for self-renewal^45–47^ and the BMP pathway is important throughout HSC ontogeny, with recent evidence indicating that the first definitive HSC which arise during development are BMP-primed^48^. This enables HSCs to sustain their myeloid–lymphoid differentiation potential as they migrate and expand within different anatomical sites during development. However, by the time the HSCs populate the BM, the majority are no longer BMP activated, which could potentially reflect cell autologous changes or the influence of the different extrinsic signals within the BM microenvironment^48^. Reactivation of the pathway in CML could therefore have important influences on stem cell behaviour, especially cell fate decisions. Cell cycle regulation is tightly linked to pluripotency and stem cell fate. Here we demonstrate that higher numbers of CML-iPSCs are in G0-G1 compared to normal iPSCs, regardless of BMP4 stimulation. This is important as this property in LSCs promotes their quiescence even in the presence of TKI therapies. Targeting the iPSC lines had no effect on cell cycle progression in the presence or absence of BMP4 stimulation. However, our results indicate CML-iPSCs are sensitive to IM treatment, reflected through their increased differentiation when treated, highlighting a role for BCR-ABL in the stemness properties of CML-iPSCs. Our protein data correlates with previous studies, demonstrating phosphorylation of STAT5 and CrKL is significantly decreased after IM treatment^49^. Here we demonstrate that IM and DOR combination treatment synergistically reduces the phosphorylation of BCR-ABL and CrkL in CML-iPSCs.

CML progenitors express higher levels of BMPRIs on their surface^16^. Our iPSC data indicate CML-iPSCs also have significantly higher levels of ALK2 and ALK3 when compared to normal iPSCs, which is sustained in the presence of BMP4 stimulation. Inhibition of the BMP pathway alone and in combination with BCR-ABL significantly decreases the expression level of AVCR1 (ALK2) in CML-iPSCs. More importantly, we demonstrate that CML-iPSCs show a greater level of sensitivity to the dual treatments, which result in loss of pluripotency and increased differentiation when compared to normal iPSCs. This was confirmed by the upregulation of key genes involved in meso-endodermal differentiation and the downregulation of important pluripotency genes including *NANOG*. These findings highlight how deregulated BMP signalling in CML could lead to an altered balance between self-renewal and differentiation.

BMP/SMAD signalling regulates early response genes through BMP-responsive elements (BRE) in their promoters, including ID1, important for development, cell cycle G1 progression and tumorigenesis^45,50^. ID1 is upregulated in CML^22,51,52^ and promotes proliferation and migration^50^. *ID1* expression is also enhanced through BCR-ABL-mediated STAT5 activation^51^. The ID1 promoter has a SRC-responsive element upstream of the translational start site, with SRC co-operating with SMAD to induce its expression^53^. Given that BCR-ABL interacts and activates SRC kinases, ID1 upregulation through BCR-ABL dependent and independent mechanisms is likely to have a key role in CML pathophysiology. Our data clearly demonstrate that IM reduces transcriptional levels of *ID1* with further reductions in the presence of LDN and DOR in both CML CD34^+^ and CML-iPSCs. This has important implications as ID1 is known to mediate its effects by downregulating *p53* and *PTEN* transcription, resulting in enhanced AKT phosphorylation and AKT-mediated canonical Wnt signalling^22^. ID1 can also enhance G1-S cell cycle progression and augments Hh and Wnt signalling through suppression of CULLIN3, an ubiquitin ligase, which targets CyclinE, GLI2 and DVL2 for degradation^54^. The BMP and Wnt pathways converge to regulate the CDX family of homeobox transcription factors, master regulators of HOX gene expression^55,56^. Given that deregulation of the Hh^13,57^ and Wnt^57^ pathways and the Hox axis^57,58^ have key roles in CML, it is tempting to speculate that ID1 could be an important orchestrator in this process (Fig. 7e).

Inhibiting type 1 BMP receptors with small-molecule inhibitors, in combination with TKIs, is a promising approach to deplete primitive CML stem/progenitor cells, especially given the emerging role of BMP pathway signalling in treatment resistance and disease relapse^16,19^. We demonstrate that combination treatment caused irreversible cell cycle arrest, increased apoptosis, reduced cell division and survival of CD34^+^ cells in CML compared to normal cells, with CML-iPSCs displaying less self-renewing potential and enhanced differentiation following therapy. Targeting pathways CML cells depend on for self-renewal especially BMP pathway is an attractive approach (Supplemental Table 1)^59–64^. Our results indicate a potential therapeutic window in CML, with intervention using BMP inhibitors in combination with TKI warranting further investigation to prevent LSCs self-renewal and improve treatment for patients in the future.

## Materials and methods

### Patient samples

Normal donors and CML samples were taken at diagnosis following informed consent in accordance with the Declaration of Helsinki, and approval of the Greater Glasgow and Clyde National Health Service. Samples were enriched for CD34^+^ cells using the CliniMACS (Miltenyi Biotec) immunomagnetic beads system. Cells were cryopreserved in 10% DMSO (Sigma-Aldrich) and 5% human albumin solution (Baxter Healthcare). SPIRIT2, a multicentre phase III randomised trial (NCT01460693; clinicaltrials.gov), in which CML patients were randomised into two groups, to compare the TKIs, IM (400 mg daily) vs Dasatinib (100 mg Daily). Sixty samples of cells were obtained from the biobank in Glasgow (30 CD34^+^ purified from peripheral blood, 30 MNC samples, 11 of which were matched, i.e. both CD34^+^ and MNC available from the same patients). Following thawing, RNA was extracted using RNAEasy Micro kits (Qiagen) or Picopure RNA isolation Kit (Thermo Fisher) to create an RNA bank of samples. Reverse transcription using Superscript III (Thermo Fisher) was then carried out to create cDNA library from all of the samples. Normal BM CD34^+^ cells were bought from Stem Cell Technologies and normal CD34^+^ from peripheral blood were obtained from the Glasgow Biobank. Six normal CD34^+^ and four normal MNC samples were used as comparators.

### iPSC generation, maintenance and characterisation

CytoTune®-iPS Sendai Reprogramming Kit (Life Technologies) was used for iPSC generation from normal CD34^+^ (Stem Cell Technologies) and treatment naive CP-CML CD34^+^ cells as per manufacturer’s instructions (Thermo Fisher). In brief, reprogramming was performed as follows: CD34^+^ cells were cultured in StemSpan (Stem Cell Technologies) media supplemented with 10% Myelocult (Stem Cell Technologies, BC, Canada), interleukin (IL)-3 (10 ng/mL), IL-6, granulocyte-colony stimulating factor (G-CSF), stem cell factor (SCF), Fms-like tyrosine kinase-3 ligand (FLT3L), (all at  100 ng/mL) (PeproTech) and 1% Penicillin/Streptomycin (Invitrogen, Paisley, UK) for 2 days prior to viral transduction. hOCT3/4, hSOX2, hKlf4, hC-MYC vectors were used at multiplicity of infection (MOI) = 6. iPSCs, once generated, were cultured in TeSR-E8 medium (Stem Cell Technologies) on matrigel (Corning)-coated Nunclon petri dishes (Fisher Scientific) in feeder-free conditions. IPSCs were passaged using EZ-Passage tool and DMEM/F12 Dulbecco’s Modified Eagle Medium (Life Technologies) twice a week. Alkaline phosphatase (APh) staining was used to assess pluripotency (Sigma-Aldrich). Live APh staining was performed using IncuCyte ZOOM Live Cell Imaging System and software (Essen Bioscience). The expression level of key pluripotency markers OCT4, SOX2, SSEA4, and/or TRA-1-60 was detected using PSC 4-Marker Immunocytochemistry Kit (Life Technologies) according to manufacturer’s instructions.

### Cell line and primary samples cell culture

K562 cells (DSMZ) were grown in RPMI 1640 (Sigma-Aldrich) with 10% foetal calf serum, 1 mM glutamine and 1% penicillin–streptomycin (Invitrogen). HS5 cells were grown in DMEM (Gibco) with 10% fetal calf serum, 1 mM glutamine and 1% penicillin–streptomycin (Invitrogen). 24 h prior to co-culture, HS5 were seeded on collagen coated 12-well plates at a cell density of 1.2 × 10^5^ cells per well.

CD34^+^-enriched normal and CML cells were thawed and cultured overnight in serum free media (SFM) (Life Technologies) supplemented with; IL-3 (20 ng/mL), IL-6 (20 ng/mL), G-CSF (20 ng/mL), SCF and FLT3L (100 ng/mL) (PeproTech). Prior to treatment with inhibitors, culture media for CD34^+^ enriched cells was changed to more physiological growth factor concentrations; IL-3 (0.2 ng/mL), IL-6 (0.2 ng/mL), G-CSF (0.2 ng/mL), SCF and FLT3L (1 ng/mL).

### Inhibitors

Overall, 100 mM stock solution of IM (Stratech) was prepared in water and stored at 4 °C; BMP pathway inhibitors LDN-193189 (LDN) (Cellagen technology) and Dorsomorphin (DOR) (Abcam) were prepared using DMSO and 10 mM stocks stored at −20 °C. Inhibitors were diluted in complete media as required. LDN interrupts the BMP pathway through inhibiting AVCR1 (ALK2) and BMPR1A (ALK3), receptor phosphorylation, whereas DOR inhibits AVCR1 (ALK2), BMPR1A (ALK3), BMPR1B (ALK6), thereby preventing phosphorylation of SMADs 1, 5 and 8, and transcription of downstream genes.

### Trypan blue exclusion cell counts and XTT bioreduction assay

Samples were diluted in Trypan Blue dye (Sigma-Aldrich) of an acid azo exclusion medium by preparing a 1:1 dilution of the cell suspension using a 0.4% Trypan Blue solution. Half maximal inhibitory concentration (IC50) of each of the BMP pathway inhibitors and IM in K562 cell line was established using XTT sodium 3′-[1-[(phenylamino)-carbonyl]-3,4-Tetrazzolium]-bis(4-methoxy-6-nitro) benzene sulphonic acid hydrate cell proliferation assay (Sigma-Aldrich) according to manufacturer’s instructions. The half maximal inhibitory concentration (IC50) of each of the BMP pathway inhibitors and IM were calculated using Prism software (GraphPad Software).

### Synergy studies using CalcuSyn

Synergy studies of either LDN or DOR in combination with IM was performed using CalcuSyn (Biosoft). The median-effect graph which is a plot of *x* = log (Dose of drug) vs *y* = log (Fa) by the dose/the fraction unaffected and dose–effect curve together with the combination index (CI) ranges for these inhibitors was used to determine synergism in targeting CML cells. CalcySyn performs multiple drug dose–effect calculations using the median-effect methods described by T-C Chou and P. Talalay^65^. The resulting combination index (CI) theorem of Chou-Talalay was used to provide a quantitative definition for additive effect (CI = 1), synergism (CI < 1), and antagonism (CI > 1) in drug combinations.

### Flow cytometry

For cell division analysis, CD34^+^ cells were stained with the proliferation dye CellTrace™ Violet (Thermo Fisher) and labelled with anti-CD34 (BD Biosciences), as recommended by manufactures protocol. For establishing a maximum point of fluorescence staining, cells were cultured in the presence of Colcemid (100 ng/mL, Invitrogen Life Technologies) to determine non-dividing cells after labelling (CTV max). All primary CD34^+^ cells were treated with IM, LDN, DOR or combinations in the presence and absence of BMP4 and cultured as described above in physiological growth factors or on the HS5 stromal cells. Annexin V/7AAD (BD Biosciences) staining was used to assess apoptosis by flow cytometry using 1 × 10^5^ cells per condition. Propidium iodide (PI) staining buffer (BD Bioscience, Oxford, UK) was used to assess cell cycle progression as per manufacturer’s protocol. To test for re-entry into cell cycle, cells were treated with inhibitor concentrations as indicated for 24 h; cells were then washed x5 with PBS prior to culture in complete media for a further 24, 48 and 72 h and PI staining performed. BMP pathway type I receptors and ALKs 2, 3 and 6 (R&D systems) were detected by intracellular flow cytometry according to the manufacturer’s instructions. A list of antibodies used is provided in Table S2.

### Fluorescence in situ hybridisation (FISH)

Detection of *BCR-ABL* fusion in CD34^+^ 38^−^ CML cells by FISH was performed as previously reported^2^.

### Protein analysis and quantitative real-time polymerase chain reaction

Whole cell lysates were prepared and SDS-PAGE western blotting was performed as described previously^2^ using PathScan Multiplex Western Cocktail primary antibody, pCRKL (Cell Signalling Technology), SHPTP2 (Santa Cruz Biotechnology), Anti-Phosphotyrosine Antibody, clone 4G10 and phospho-SMAD1/5/8 (Millipore). Immunofluorescent staining was performed on Poly-l-lysine slides, cells were fixed with 4% formaldehyde for 10 min and permeabilised with 0.5% Triton-X100 in PBS for 15 min. Following incubation with 5× blocking solution for 2 h, proteins were detected using anti-SMAD4 (Millipore) and phospho-SMAD1/5/8 primary antibodies overnight at 4 °C. Following detection using the corresponding conjugated secondary antibody, slides were then visualised under Zeiss epifluorescence microscope and images analysed using AxioVision Rel.4.8 microscopy software. A list of antibodies used is provided in Table S3. Quantitative real-time polymerase chain reaction (qRT-PCR) was undertaken using the Fluidigm BioMark HD System and TaqMan (Applied Biosystems) gene expression assays, as per manufacturer’s instructions. Eight low/medium copy number reference genes were used in the analysis. A list of primers used is provided in Table S4 with reference genes shaded in grey.

### Assessment of hematopoietic progenitors

Colony-forming cell (CFC) assays were performed as described by Holyoake et al.^1^ Overall, 4 × 10^3^/mL cells from each treatment arm were re-suspended in 300 µL SFM and inoculated in 3 mL Methocult H4034 (Stem Cell Technologies). This was then split into two 35 mM dishes and incubated in a humidified incubator. CFCs were counted on day 11.

### Statistical analyses

Average responses from at least three independent experiments are shown (mean ± SEM). Statistical analysis was performed using GraphPad Prism 6 (GraphPad Software Inc.), using Anova, Welch’s test, Student’s paired *t*-test for matched samples, and Mann–Whitney *U* test for unpaired samples.

## Electronic supplementary material

Supplemental material

Supplementary Figure Legends
