# Supplementary material for: Chronic myeloid leukaemia cells require the bone morphogenic protein pathway for cell cycle progression and self-renewal
Source: Cell Death Dis. 2018 Sep 11;9(9):927. doi: 10.1038/s41419-018-0905-2 (PMC6134087; doi:10.1038/s41419-018-0905-2)
Supplement: Supplementary file 1 — Supplemental material [file 41419_2018_905_MOESM1_ESM.docx]

**Supplemental material**

**Supplemental Table 1**

| **FACS antibodies** |  |
| --- | --- |
| **Antibody** | **Fluorochrome** |
| CD34 (BD Bioscience, Oxford, UK) | APC |
| 7AAD (BD Bioscience, Oxford, UK) | PE |
| Annexin V (BD Bioscience, Oxford, UK) | FITC |
| Anti-human BMPR-IA (ALK3) (R&D systems, Abingdon, UK) | PE |
| Anti-human BMPR-IB (ALK6) (R&D systems, Abingdon, UK) | APC |
| Donkey anti-goat IgG secondary (H+L) (R&D systems, Abingdon, UK) | PE |
| Human activing R1A (ALK2) antigen affinity-purified polyclonal goat IgG (R&D systems, Abingdon, UK) | - |

**Supplemental Table 2**

| **Western blot/ IF antibodies** |  |  |
| --- | --- | --- |
| **Antibody** | **Band size** | **Dilution** |
| Alexa Fluor 488 green anti-rabbit immunofluorescence secondary antibody (Life Technologies, NY, USA) | - | 1:300 |
| Alexa Fluor 594 red anti-mouse immunofluorescence secondary antibody (Life Technologies, NY, USA) | - | 1:300 |
| Anti-mouse IgG horseradish peroxidise (HRP) linked secondary antibody (Life Technologies, NY, USA) | - | 1:10000 |
| Anti-Phosphotyrosine Antibody, clone 4G10 (Millipore, Hertfordshire, UK) | - | 1:10000 |
| Anti-rabbit IgG horseradish peroxidise (HRP) linked secondary antibody (Dako, Denmark) | - | 1:10000 |
| P. SMAD1/5/8 primary antibody (Cell signalling technology, MA, USA) | SMAD1: 52-56 kDa  SMAD5: 52 kDa  SMAD8: 52 kDa | 1:1000 |
| PathScan Multiplex Western Cocktail primary antibody (Cell signalling technology, MA, USA) | P-Bcr-Abl: 210 kDa  P-Stat5: 90 kDa  P-CrkL: 39 kDa | 1:1000 |
| SH-PTP2 primary antibody (Santa Cruz Biotechnology, Texas, USA) | 70 kDa | 1:2000 |
| SMAD4 primary antibody (Santa Cruz Biotechnology, Texas, USA) | 61 kDa | 1:1000 |

**Supplemental Table 3: Primer sequences**

| **Gene** | **Forward (5'>3')** | **Reverse (5'>3')** |
| --- | --- | --- |
| ***ACTC1*** | GCAGTGCTATCCCTGTATGCT | GGTAGTCAGTGAGGTCCCGA |
| ***ACVR1 (ALK2)*** | GCCAAGGGGACTGGTGTAAC | GAGAATAATGAGGCCAACCTCCA |
| ***ACVR1B (ALK4)*** | GACATTGCCCCGAATCAGAGG | GCCCGAGGGCATAAATATCAGC |
| ***ACVR1C (ALK7)*** | ATGTGACCGCCTCTGGATCT | TGCCACACCTCACCAAATCT |
| ***ACVR2A*** | ACCATGGCTAGAGGATTGGC | GCCAACCCAAAGTCAGCAAT |
| ***ACVR2B*** | CTGCAACGAACGCTTCACTC | CAGGACGATGAGGGAAAGGC |
| ***ALPL*** | GCAGCCACTGAGCGTTCC | GGGTGGCATGGTTCACTCTC |
| ***ATM*** | CGGAGCTGATTGTAGCAACATACTA | CAGATAGAGCCTGAAGTACACAGAG |
| ***ATP5B*** | TCCATCCTGTCAGGGACTATG | ATCAAACTGGACGTCCACCAC |
| ***ATR*** | CAGCTCTCTATGAAGGCCATTCAA | GTTCTACTGTTTCACTGTCTGTTGC |
| ***B2M*** | TTGTCTTTCAGCAAGGACTGG | ATGCGGCATCTTCAAACCTCC |
| ***BMP2*** | AGACCTGTATCGCAGGCACT | CCACTCGTTTCTGGTAGTTCTTCC |
| ***BMP4*** | CAGCACTGGTCTTGAGTATCCT | AGCAGAGTTTTCACTGGTCCC |
| ***BMP7*** | CAGGCCTGTAAGAAGCACGA | TGGTTGGTGGCGTTCATGTA |
| ***CCNA1*** | CTCGTAGGAACAGCAGCTATGC | GCTAGAACTTTCAGAAGCAAGTGTTC |
| ***CCNA2*** | ACCCTGGAAAGTCTTAAGCCT | GTGTCTCTGGTGGGTTGAGG |
| ***CCNB1*** | CAGCTCTTGGGGACATTGGTAAC | ACTGGCACCAGCATAGGTACC |
| ***CCND1*** | TGCATCTACACCGACAACTCC | CGGATGATCTGTTTGTTCTCCG |
| ***CCND2*** | CCGCAGTGCTCCTACTTCAA | GCCAAGAAACGGTCCAGGTA |
| ***CCND3*** | CCTCCTACTTCCAGTGCGTG | AGGCCAGGAAATCATGTGCA |
| ***CCNE1*** | CAACGTGCAAGCCTCGGA | CAAAGTGCTGATCCCTTAAGTATGTC |
| ***CCNE2*** | ATCCTTCACCTTTGCCTGATTT | CCTCATCTGTGGTTCCAAGTCA |
| ***CD34*** | TTCCTGATGAATCGCCGCAG | GTTCACACTGGCCTTTCCCT |
| ***CDC25A*** | GTCTAGATTCTCCTGGGCCATTG | CAGAATGGCTCCTCTTCAGAGC |
| ***CDC25B*** | GGATTTGTGGACATCCTAGAGAGT | ACTTGCTGTACATGACGAGGT |
| ***CDC25C*** | CACTCAGCTTACCACTTCTGCAG | GGGCTACATTTCATTAGGTGCTGG |
| ***CDK1*** | ATGAAGTGTGGCCAGAAGTG | CAGAAATTCGTTTGGCTGGATCA |
| ***CDK2*** | GCCTGATTACAAGCCAAGTTTCC | TCCGCTTGTTAGGGTCGTAGT |
| ***CDK4*** | CCCATCAGCACAGTTCGTGA | AACACCAGGGTTACCTTGATCTC |
| ***CDK5*** | TCTTCCAGCTACTAAAAGGGCT | CAATTTCAGCTCCCCATTCCTG |
| ***CDK6*** | CCGAAGTCTTGCTCCAGTCC | GTTGATCAACATCTGAACTTCCACG |
| ***CDKN1A*** | TCTTGTACCCTTGTGCCTCG | CGGCGTTTGGAGTGGTAGAA |
| ***CDKN1B*** | GGCTAACTCTGAGGACACGC | TGAGTAGAAGAATCGTCGGTTGC |
| ***CHEK1*** | CCATCAGCAAGAATTACCATTCCAG | CTGGGAGACTCTGACACACC |
| ***CHEK2*** | ATGAGAACCTTATGTGGAACCCC | GCTCAGAGAAAGGTGGATACCC |
| ***CITED2*** | GCGAGCACATACACTACGGC | CCATGAACTGGGAGTTGTTAAACC |
| ***COL1A1*** | GCTTCACCTACAGCGTCACT | AAGCCGAATTCCTGGTCTGG |
| ***COL2A1*** | TCCAGCAAACGTTCCCAAGA | ATCTGGACGTTGGCAGTGTT |
| ***CYC1*** | ACTGCGGGAAGGTCTCTACTT | GGGTGCCATCGTCAAACTCTA |
| ***CYCD1*** | TGCATCTACACCGACAACTCC | CGGATGATCTGTTTGTTCTCCG |
| ***E2F1*** | GGACCTGGAAACTGACCATCAG | CAGTGAGGTCTCATAGCGTGAC |
| ***E2F2*** | CTCTCTGAGCTTCAAGCACCTG | CTTGACGGCAATCACTGTCTGC |
| ***E2F3*** | AGCGGTCATCAGTACCTCTCAG | TGGTGAGCAGACCAAGAGACGT |
| ***E2F4*** | GCTGACACCCTAGCTGTACG | AATCTCCCGGGTATTGCAGC |
| ***E2F5*** | ACCTGATGACCTCACACAGCCTTC | GGGGTAGGAGAAAGCCGTAAAAG |
| ***E2F6*** | GAGACCCCATCAACGTGGAG | CCAGCGATACATCAAAACGAGG |
| ***ENOX2*** | GAGCTGGAGGGAACCTGATTT | CACTGGCACTACCAAACTGCA |
| ***GAPDH*** | ACGGATTTGGTCGTATTGGG | ATTTTGGAGGGATCTGCTC |
| ***ID1*** | CGGAATCCGAAGTTGGAACC | GACACAAGATGCGATCGTCC |
| ***INHBA*** | AAGTCGGGGAGAACGGGTAT | GGTCACTGCCTTCCTTGGAA |
| ***KLF4*** | GAACTGACCAGGCACTACCG | TTCTGGCAGTGTGGGTCATA |
| ***MESP1*** | GCAGGCGATGGAGCCAAG | CAGTTGTCCCTTGTCACTTGGG |
| ***MYC*** | GACTCTGAGGAGGAACAAGA | TTGGCAGCAGGATAGTCCTT |
| ***NANOG*** | GCTACAAACAGGTGAAGACC | GCTCCAGGTTGAATTGTTCC |
| ***NCAM1*** | CAGCGTTGGAGAGTCCAAATTC | ATTCCACACCACTGAGATCCG |
| ***OCT3/4 (POU5F1)*** | GGAAGGTATTCAGCCAAACG | CTCGATACTGGTTCGCTTTC |
| ***PODXL*** | TACCCTGCCAGAGACCATGAG | CTGTGTCTGTGTCTCAAGATCCTC |
| ***REST*** | CACTGGAGGAAACATTTAAGAAACC | GACAAAGTTCACATTTATATGGGCG |
| ***RNF20*** | GGTGTCTCTTCAACGGAGGAA | TAGTGAGGCATCATCAGTGGC |
| ***RUNX1*** | AGCGAGATTCAACGACCTCA | TAGGACTGGTCATAGGACCA |
| ***RUNX2*** | AACAAGACCCTGCCCGTGG | CATTCAGCAGAGGCATTCCGG |
| ***SMAD1*** | GCTGCTCTCCAATGTTAACCG | CACTAAGGCATTCGGCATACAC |
| ***SMAD2*** | CCACGGTAGAAATGACAAGAAGG | GATTACAATTGGGGCTCTGCAC |
| ***SMAD3*** | GTCTGCGTGAATCCCTACCAC | GGGATGGAATGGCTGTAGTCG |
| ***SMAD4*** | GGGTCAACTCTCCAATGTCCAC | GTCACTAAGGCACCTGACCC |
| ***SMAD5*** | TGGGTCAAGATAATTCCCAGCCT | GGCTCTTCATAGGCAACAGGC |
| ***SMAD6*** | CTCCCTACTCTCGGCTGTCT | AGAATTCACCCGGAGCAGTG |
| ***SMAD7*** | CCATCACCTTAGCCGACTCT | CCAGGGGCCAGATAATTCGT |
| ***SMAD8*** | CTTATCATGCCACAGAAGCCTCT | GCTCCTCGTAACAAACTGGTCG |
| ***SMURF1*** | ATGCAGTTCGTGGCCAGATA | CAGGCCCGGAGTCTTCATAC |
| ***SMURF2*** | GACAGGATCCTCTCGAGTGC | AGCTTTCATAGGGTGGAATGTCT |
| ***SNAI1*** | CGGAAGCCTAACTACAGCGA | GGTGGGGTTGAGGATCTCC |
| ***SNAI2*** | AAGATGCCGCGCTCCTTC | TGACAGGCATGGAGTAACTCT |
| ***SNAI3*** | GAAAACGCACTCCAGCCAC | GTCACCGGGCACAGAAGG |
| ***SOX2*** | GCCCTGCAGTACAACTCCAT | GACTTGACCACCGAACCCAT |
| ***SOX4*** | GTTTAAACCACTGGATCTATCTAAATGCC | CACATATGCTATCATCATGCCATAAGAC |
| ***TGFB1*** | CGACTCGCCAGAGTGGTTATC | GTTATCCCTGCTGTCACAGGAG |
| ***TGFBR1*** | CGTTCGTGGTTCCGTGAGG | TAATCTGACACCAACCAGAGCTG |
| ***TP53*** | CAGTCAGATCCTAGCGTCGA | TGTTCAATATCGTCCGGGGA |
| ***TUBB3*** | AGCGTCTACTACAACGAGGC | CAGGCCTGAAGAGATGTCCA |
| ***TYW1*** | ATTGTCATCAAGACGCAGGGC | GTTGCGAATCCCTTCGCTGTT |
| ***UBE2D2*** | CCATGGCTCTGAAGAGAATCC | GATAGGGACTGTCATTTGGCC |

**Supplemental Table 4**

| **Inhibitor** | **Mechanism of action** | **Stage of study** |
| --- | --- | --- |
| **LDN-193189^19,20,59^** | Targeting ALKs 2,3 | Under development for the treatment of patients with progressive fibrodysplasia ossificans (FOP) (National centre for advancing translational sciences) |
| **Dorsomorphin^19,59^** | Targeting ALKs 2,3,6 | Under development for the treatment of patients with FOP (National centre for advancing translational sciences) |
| **llama-derived antibodies ^60^** | Lacking light chains, targeting interactions in the BM niche | - |
| **Primozide, SJB2-043, ML323, GW7647^61,62^** | Small molecule inhibitors of USP1, ubiquitin-specific protease which de-ubiquinate Id1, thereby rescuing it from proteosomal degradation | - |
| **Lorlatinib (PF-06463922)^63^** | Small molecule inhibitor of ROS1 and ALKs | Phase1/2 trial for patients with ALK-positive or ROS1 positive advanced non-small cell lung cancer (ClinicalTrials.gov Identifier: NCT01970865) |
| **Saracatinib (AZD0530)^64^** | Small molecule inhibitor of SCR/ABL kinases but has efficacy in targeting ALKs, strongly inhibiting the kinase activity of ALKs 1, 2 & 3 with IC50’s in the 3-30nM range and moderately inhibiting ALKs 4, 5 & 6 with IC50’s in the 300-800nM range, also has the potential to be effective against Id1 as it would prevent transcription initiation through the BRE- and SRC-responsive element | Effective at inhibiting Philadelphia chromosome positive CML cell lines and BaF3 cells expressing the E255K and Y253F but not the T315I BCR-ABL mutations |

59. Sanvitale, C.E. et al. A new class of small molecule inhibitor of BMP signaling. *PLoS One* **8,** e62721 (2013).

60. Calpe, S., Correia, A.C., Sancho-Serra Mdel, C., Krishnadath, K.K. Comparison of newly developed anti-bone morphogenetic protein 4 llama-derived antibodies with commercially available BMP4 inhibitors. *MAbs* **8,** 678-688 (2016).

61. Mistry, H. et al. Small-molecule inhibitors of USP1 target ID1 degradation in leukemic cells. *Mol Cancer Ther* **12,** 2651-2662 (2013).

62. Das, D.S. et al. Blockade of Deubiquitylating Enzyme USP1 Inhibits DNA Repair and Triggers Apoptosis in Multiple Myeloma Cells. *Clin Cancer Res* **23,** 4280-4289 (2017).

63. <http://press.pfizer.com/press-release/pfizers-next-generation-alkros1-inhibitor-lorlatinib-granted-breakthrough-therapy-desi>

64. <https://openinnovation.astrazeneca.com/saracatinib-azd0530.html>

**Supplemental Figure 1**

**Supplemental Figure 2**

**Supplemental Figure 3**

**E. Summary of apoptosis data**

**Supplemental Figure 4**

**Supplemental Figure 5**
