# Supplementary material for: Chronic myeloid leukaemia cells require the bone morphogenic protein pathway for cell cycle progression and self-renewal
Source: Cell Death Dis. 2018 Sep 11;9(9):927. doi: 10.1038/s41419-018-0905-2 (PMC6134087; doi:10.1038/s41419-018-0905-2)
Supplement: Supplementary file 2 — Supplementary Figure Legends [file 41419_2018_905_MOESM2_ESM.docx]

**Figure S1.** The interaction between the BMP/TGFβ pathway components were extracted from Metacore functional analysis software and entered into Cytoscape network analysis program to obtain a network map. The CP-CML CD34+ gene profiling was then incorporated and genes up- and down-regulated were mapped onto the network. This figure illustrates the final results of this network analysis. There is a close interaction between BMP pathway and cell cycle genes controlling G1-S transition with a large number of these genes being significantly deregulated. BMP-controlled cell cycle genes are important in CP-CML. Red nodes: statistically significant up-regulation, blue nodes: statistically significant down-regulation, red arrows: activation, blue dotted arrows: inhibition.

**Figure S2 The effect of IM and BMP inhibitors on cell cycle progression in the presence of BMP4 and following co-culture with HS-5.** **A i-ii.** Propodium iodide (PI) analysis of cell cycle progression of normal and CP CML CD34^+^samples treated with IM, BMP inhibitors and the combination of both (IM = 1µM, LDN = 1µM, DOR = 2.5μM) at 72hr. Data show a significant increase in the number of cells in Sub-G0 in all single and dual treatments of normal and to a higher extent for CML samples additionally treated with BMP4 (n=3) **B i-ii.** Analysis of cell cycle progression of normal and CP CML CD34^+^ in co-culture with HS-5 do not reveal any significant differences across all cell cycle phases. **C.** Representative histogram overlay of one biological replicate for each, normal and CP CML CD34^+^ cells treated with IM and BMP inhibitors in absence or presence of BMP4 and in co-culture with HS-5. **D i-ii.** Comparison amongst absence and presence of BMP4 and co-culture conditions reveal a significant increase in Sub-G0 across all samples with single and dual DOR treatment. Additionally, all CP CML CD34^+^ samples display a significant increase for single and dual treatment of LDN (n=3). There was no significant difference for other phases of the cell cycle in the absence or presence of BMP4 across all stages of cell cycle progression. Cell cycle PI data are expressed as mean ± standard deviation and were compared using ANOVA ****p < 0.0001, *** p 0.001 to 0.0001, ** p 0.01 to 0.001, *p 0.05 to 0.01.

**Figure S3 IM and BMP inhibitors induce apoptosis in CML cells cultured with BMP4. A-D.** AnnexinV/ 7AAD apoptosis analysis of normal and CP CML CD34^+^ cells treated with IM, BMP inhibitors and in combination (IM = 1µM, LDN = 1µM, DOR = 2.5μM, n=3) at 72hr. **A.** Representative example of gating strategy for apoptosis data analysis. After excluding cell debris, we excluded duplets and finally based our gates on FMO (Fluorescence minus one) controls for AnnexinV and 7AAD. **B.** Example dot plots of one biological replicate for each sample of normal and CP CML CD34^+^ cells cultured with BMP4. **C.** Normal and CP CML CD34^+^ cells co-cultured with HS-5 did not reveal any significant difference in apoptosis amongst IM and BMP inhibitors (n=3). **D i-ii.** Comparison of absence and presence of BMP4 and co-culture conditions regarding treatments with DOR and BMP inhibitors in combination with IM, reveal a significant increase in viable cells and a significant decrease of cells in late apoptosis when co-cultured with HS-5 (n=3). There was no significant difference in the absence or presence of BMP4 in viable or apoptotic cell. Apoptosis data are expressed as mean ± standard deviation and were compared using ANOVA ****p < 0.0001, *** p 0.001 to 0.0001, ** p 0.01 to 0.001, *p 0.05 to 0.01. **E.** summarises the apoptosis data and shows the % of cells in each gate for all the experimental conditions.

**Figure S4 Single treatment of DOR and dual treatments with BMP inhibitors and IM inhibit cell proliferation of CML CD34^+^ cells. A.** CellTrace™ Violet (CTV) proliferation analysis of normal and CP CML CD34^+^ cells treated with IM, BMP inhibitors, the combination (IM = 1µM, LDN = 1µM, DOR = 2.5μM, n=3) in co-culture with HS-5 at 72hr. **i.** Results for normal CD34^+^ co-cultured cells display a difference in fold change for single and dual treatment of DOR. **ii.** The same results but with a higher fold change were observed with CML CD34^+^ co- cultured cells. **iii.** The number of CD34^+^ cells decreases with IM and BMP inhibitor treatment in normal and CP CML cells (n=3). **B-D.** Representative histograms of one biological replicate of each, normal and CP CML CD34+ cell divisions with IM and inhibitor treatments in absence or presence of BMP4 and in co-culture. All histograms are displayed in modal. **E.** Data for all CTV proliferation analyses. **i.** Percentages of normal and CP CML cells in each division. **ii.** Percentages of normal and CP CML CD34^+^ cells in each division. **iii.** Total number of normal and CP CML CD34^+^ cells in each division.

**Figure S5 A.** Cell cycle analysis of normal and CML-iPSCs treated with IM, BMP inhibitors, and the combination using PI staining in the **i.** absence and **ii.** Presence of BMP4 stimulation. There was no change in cell cycle progression following any of the treatment arms. **B.** **i.** AP colony counts detecting pluripotency in normal and CML-iPSCs treated with IM, BMP inhibitors or the combination (1μM IM, 1μM LDN, 2.5μM DOR) at 96hr. Red lines and red stars correspond to statistical analysis between IM single treatment and dual inhibition. **ii.** Examples of differentiated and pluripotent iPSC colonies. Red colour intensity indicates the level of AP expression with the colonies containing more pluripotent cells in darker red. Dual inhibition with IM and either of the BMP inhibitors in CML-iPSCs has a synergistic effect on reducing the “stemness” of CML colonies. Data are expressed as mean ± standard deviation and were compared using the unpaired Student t-test, ** p 0.01 to 0.001, *p 0.05 to 0.01, (n=3 normal and n=3 CML iPSC samples).
